# Investigation and computational prediction of gating pore currents in Na_V_1.2 mutations across clinical phenotypes

**DOI:** 10.1093/pnasnexus/pgag230

**Published:** 2026-07-02

**Authors:** Ahmed Eltokhi, Eslam Elhanafy, Jing Li, Tamer M Gamal El-Din

**Affiliations:** Department of Biomedical Sciences, School of Medicine, Mercer University, Columbus, GA 31901, USA; Department of Biomolecular Sciences, School of Pharmacy, University of Mississippi, Oxford, MS 38677, USA; Department of Biomolecular Sciences, School of Pharmacy, University of Mississippi, Oxford, MS 38677, USA; Department of Pharmacology, University of Washington, Seattle, WA 98195, USA

**Keywords:** voltage-gated sodium channels, gating pore currents, developmental and epileptic encephalopathy, benign familial neonatal-infantile seizures, autism spectrum disorder

## Abstract

Voltage sensor domains (VSDs) are integral and common regions within voltage-gated ion channels that play a crucial role in cellular signaling and excitability. Each VSD comprises four transmembrane segments (S1–S4) per domain. The transmembrane segment S4 contains positively charged amino acid residues at every third position, which serve as the channel's gating charges. Over the last decade, it has been shown that these gating charges are the target of many pathological mutations. This study investigates three gating-charge mutations in domain I of the Na_V_1.2 channel (R220G, R223I, and R223Q) identified in patients with distinct clinical phenotypes. The three mutations showed differential gating pore current (*I*_gp_) amplitudes with distinct voltage dependence. To elucidate the molecular basis for the differential *I*_gp_ observed among these mutants, we performed microsecond-scale molecular dynamics (MD) simulations and a comprehensive analysis of the state-dependent interaction network within VSD_I_ across WT and mutant conformational states. The results showed that cation–π interactions between the S4 gating charges and the hydrophobic residue in S2 are the main forces that prevent VSD from leaking *I*_gp_. Hydrophobic interactions represent the second line of defense against VSD leaking, preventing gating pore formation by maintaining structural integrity across different conformations. Salt-bridge interactions and hydrogen bonds are important stabilizing forces; however, within the VSD, their contribution appears to be less significant than that of cation–π and hydrophobic interactions. Predicting and identifying *I*_gp_ as a potential shared pathophysiology across diverse clinical presentations may guide the design of targeted therapeutic strategies.

Significance statement Voltage-gated ion channels are fundamental to electrical signaling in nerves, muscles, and the heart. While their central pores have been extensively studied, abnormal ion conduction through the voltage sensor domain (VSD)—known as gating pore currents (*I*_gp_)—has emerged as a critical but underexplored mechanism of channel dysfunction. *I*_gp_ arise from mutations that create pathological leak pathways, disrupting cellular ion homeostasis and membrane excitability. *I*_gp_ are now recognized as key contributors to inherited channelopathies, including periodic paralysis, cardiac arrhythmias, and neurological disorders. Studying *I*_gp_ provides unique insight into VSD structure–function relationships, reveals previously unappreciated mechanisms of disease, and may help identify potential therapeutic targets. Understanding the molecular basis of *I*_gp_ is therefore essential for advancing fundamental ion channel biology and precision medicine approaches to channelopathies.

## Introduction

Voltage-gated ion channels are transmembrane proteins that generate action potentials, regulate neurotransmitter release, and govern intrinsic excitability. As viewed from the extracellular side, they share a common structural motif: a central pore formed by modules contributed by four domains (1, 2). Each domain contains six transmembrane segments (TM), where the first four TM (S1–S4) form the voltage-sensing module, and the remaining two TM (S5 and S6) create a central ion pore (3). These four voltage sensor domains (VSDs) are symmetrically located on the periphery. They sense changes in membrane potential via the electrostatic force exerted on their gating charges, and they initiate conformational changes that first lead to channel activation and eventually to inactivation.

The S4 segment of voltage-gated ion channels bears four or more conserved arginine (Arg, R) or lysine (Lys, K) residues that serve as gating charges in the voltage sensor and drive electrical excitation of these channels. Mutation of one or more of the gating-charge residues into a smaller amino acid with an uncharged side chain was previously shown to induce a cation current leak, known as omega current (*Ι_ω_*) or gating pore current (*I*_gp_), and was initially observed in the *Shaker* potassium channel (4, 5) (For a recent review, see Ref. (6)). An inward *I*_gp_ in the resting state was then detected in double mutations of R1 and R2 of the Na_V_1.2 channel, while double mutation of R2 and R3 caused an outward *I*_gp_ in the activated state (7). In the voltage-gated Na_V_1.4 channel, mutations affecting the outermost arginine gating charges (R1 or R2) within the VSD have been linked to hypokalemic periodic paralysis (8–10). These mutations induce an aberrant inward *I*_gp_ during the resting state (8–10).

In contrast, mutations of the third arginine gating charge (R3) cause normokalemic periodic paralysis owing to outward cationic *I*_gp_ in activated/inactivated states (11). The same phenomenon was observed in bacterial sodium channels, Na_V_Ab and NaChBac, confirming the conservation of the VSD structure from archaebacteria to humans (3, 12–14). The structural basis of *I*_gp_ in hypokalemic and normokalemic periodic paralysis was elucidated in 2018 through crystal structures of gating pore mutations introduced into Na_V_Ab (13). For Na_V_1.5, mutations in the Arg gating charges were found in patients with arrhythmia and dilated cardiomyopathy, inducing inward and outward *I*_gp_ depending on the mutation's location (15–17). *I*_gp_ induced by mutations in the Arg gating-charge residues in domains I and III of Ca_V_1.1 was also associated with hypokalemic periodic paralysis (18, 19). In addition to the aforementioned studies measuring cation *I*_gp_, proton *I*_gp_ can be induced in Arg-to-His mutations through a Grotthuss hopping mechanism. This process involves proton transfer facilitated by the reversible protonation and deprotonation of His at different pH (20), as shown in potassium (20), sodium (21–24), and calcium channels (25).

In 2021, we identified the first potential pathogenic link between *I*_gp_ and autism spectrum disorder (ASD) in K_V_7.2 and K_V_7.3 potassium channels (26). ASD-related mutations of Arg residues, R1 or R2, caused inward *I*_gp_ in the resting state, which was blocked upon voltage sensor activation. In contrast, mutations in R4 were nonconductive in the resting state but conducted outward *I*_gp_ in the activated and inactivated states. Although the amplitude of *I*_gp_ was <4% of the central pore current, it severely affected neuronal excitability, leading to hypoexcitability in dopaminergic neurons. This observation highlights the impairment in information processing in the brains of ASD patients (26). The association between *I*_gp_ and ASD was further confirmed by another study in Na_V_1.2, which revealed that ASD-related mutations in domains II and IV also induced *I*_gp_ (24). The in silico modeling of the gating pore current effect in that study suggests hyperexcitability of cortical neurons, highlighting the diverse possible impacts of *I*_gp_ on neuronal properties (24). All these studies highlight *I*_gp_ as a shared pathophysiological mechanism across disorders involving multiple voltage-gated ion channels (for a recent review, see Ref. (6)). In addition, it has been shown, functionally and computationally, that the same amino acid substitutions at equivalent gating-charge positions in different VSDs within the same channel exhibit differential gating properties and induction of *I*_gp_ (21, 27). These observations prompted us to investigate the microdeterminants of eliciting *I*_gp_ through exploring additional disorders in which *I*_gp_ may contribute to pathogenesis.

Mutations in the brain Na_V_1.2 sodium channel cause a wide array of phenotypes ranging from benign familial neonatal-infantile seizures (BFNIS) and developmental and epileptic encephalopathy (DEE) to neurodevelopmental disorders such as ASD and intellectual disability (ID) (28). Therefore, in this study, we combined functional and computational approaches to investigate whether mutations in the Arg gating charges of Na_V_1.2 linked to other clinical phenotypes can also induce *I*_gp_, and, if so, to uncover the distinct molecular mechanisms underlying their different clinical phenotypes. Using the ClinVar database (29), three mutations targeting the Arg gating charges of the S4 segments in domain I (R2: R220G, DEE) (R3: R223I, ASD) and (R3: R223Q, BFNIS) were identified. Expressing these mutations in HEK293 cells using our enhanced method of expression (a combination of the mammalian baculovirus system (BacMam) and thymidine treatment) (30) revealed different amplitudes of inward *I*_gp_ at negative potentials, along with mutation-specific alterations in activation, inactivation, and recovery from fast inactivation. Our results indicate that *I*_gp_ is associated with epilepsy-related disorders, and thus its amplitude and voltage range may have distinctive effects on neuronal functions. To elucidate the structural basis for the differential *I*_gp_ observed among the Na_V_1.2 gating-charge mutants, we performed microsecond-scale molecular dynamics (MD) simulations and a comprehensive analysis of the state-dependent interaction network within VSD_I_ across different conformational states for WT and mutant trajectories. This analysis highlights the key (electrostatic, cation–π, and hydrophobic) interactions that contribute to the conformational stability of the VSD. Disruption of these interactions by missense mutations facilitates aberrant gating pore opening. This study establishes a mechanistic framework for predicting and investigating *I*_gp_ in voltage-gated ion channels caused by missense mutations targeting the VSD.

## Results

### Gating-charge mutations in domain I of Na_V_1.2 induce differential inward gating pore currents

We identified three missense mutations in Arg gating-charge residues of the S4 TM segment in domain I of the Na_V_1.2 channel (R220G, R223I, and R223Q) using the ClinVar database. Based on our previous experiments in Na_V_1.2, *I*_gp_ are estimated to be in the picoampere range and around 0.1% of the central pore current at −100 mV (24), which were difficult to be assessed using standard methods of transfection (31, 32). Therefore, we achieved a high Na_V_1.2 current density in HEK293 cells using our established expression method, combining the mammalian baculovirus system (BacMam) and thymidine treatment to synchronize the cell cycle at the G1/S boundary (30). This method achieved high-level expression of R220G, R223I, and R223Q mutant channels, as indicated by increased peak amplitudes of the central pore current, averaging ∼20 nA (Fig. S1).

**Figure 1:**
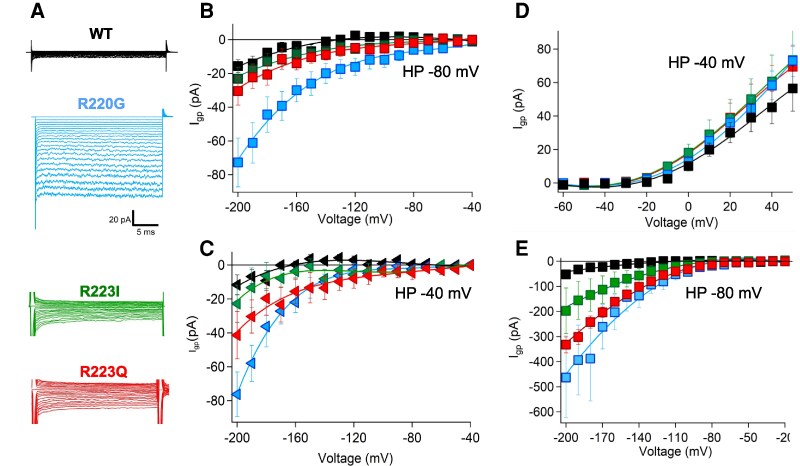
Inward gating pore currents (*I*_gp_) induced by Na_V_1.2 mutations. A) Representative traces of *I*_gp_ conducted by Na_V_1.2 (R220G, R223I, and R223Q) compared to nonspecific leak current by WT. B) Current–voltage (I–V) curves of Na_V_1.2 mutants (R220G, R223I, and R223Q) and leak current of WT Na_V_1.2. Cells were held at −80 mV, and 20 ms voltage steps from −200 mV to −10 mV in 10 mV increments were applied. Inward *I*_gp_ carried by Na^+^ started at −60 mV for R220G, while it started at −100 mV for both R223I and R223Q. *n* = 7, 8, 9, and 8 for R220G, R223I, R223Q, and WT, respectively. C) I–V curves of Na_V_1.2(R220G, R223I, and R223Q) and nonspecific leak current for Na_V_1.2(WT) when cells were held at −40 mV. Inward *I*_gp_ carried by Na^+^ were induced by R220G, R223I, and R223Q starting at −80 mV and increased at negative potentials. *n* = 5, 7, 8, and 6 for R220G, R223I, R223Q, and WT, respectively. Error bars indicate SEM. The extracellular patch-clamp solution contained: 140 mM NaCl, 1 mM CaCl_2_, 1 mM MgCl_2_, and 10 mM HEPES. D) R220G, R223I, and R223Q did not produce outward *I*_gp_ when cells were held at −40 mV. E) In the absence of CaCl_2_ and MgCl_2_ in the extracellular solution, the amplitudes of *I*_gp_ induced by R220G, R223I, and R223Q were increased compared to the presence of 1 mM CaCl_2_ and MgCl_2_ in the extracellular solution (B). *n* = 6, 6, 5, and 7 for R220G, R223I, R223Q, and WT, respectively. The extracellular patch-clamp solution contained: 140 mM NaCl and 10 mM HEPES. HP, holding potential.

To mimic the physiological ionic concentrations in the cerebrospinal fluid (CSF) bathing neurons in the brain, we used an extracellular solution containing 140 mM NaCl, 1 mM CaCl_2_, 1 mM MgCl_2_, and 10 mM HEPES, and we added 1 μM tetrodotoxin to our external solution to block the ion permeation pathway through the central pore. HEK293 cells were held at −120 mV for 10 min to allow Na_V_1.2 channels to recover from slow inactivation. To measure *I*_gp_, cells were held at −80 mV, and pulses were applied from −200 mV to −10 mV in 10-mV increments for a duration of 20 ms (Fig. 1A, B). Cells expressing the Na_V_1.2 (R220G, light blue traces) mutant channel revealed a voltage-dependent inward leak current representing *I*_gp_ and carried mainly by Na^+^ starting at −60 mV (*P*≤0.001) and increased at more negative potentials compared to WT, which showed only nonspecific leak current (Fig. 1A, B). Comparing *I*_gp_ amplitude to the central peak current amplitude of R220G channel indicates that *I*_gp_ is around 0.06 and 0.1% at −60 mV and −100 mV, respectively. These results are consistent with our previous study, revealing that *I*_gp_ induced by gating-charge mutations in domains II (R853Q) and IV (R1626Q and R1629H) of the Na_V_1.2 channel is ∼0.1% of the amplitude of the central pore current at −100 mV (24). However, the voltage onset of *I*_gp_ in R220G was more positively shifted (−60 mV) compared to R853Q, R1626Q, and R1629H, first appearing at −80/−90 mV (24).

For gating-charge mutations in R3, R223I (green traces) and R223Q (red traces), *I*_gp_ were subtle and first appeared at −100 mV (Fig. 1A, B). The *I*_gp_ amplitudes were ∼0.02 and 0.03% of the amplitude of central pore current for R223I and R223Q, respectively. These results indicate that the R2 mutation of domain I induces 4- to 5-fold inward *I*_gp_ compared to the R3 mutations, suggesting that R2 occupies the hydrophobic constriction site (HCS) at our holding voltage of −80 mV, while R3 is located in the intracellular water crevice. To test this hypothesis, we repeated the experiment using the same pulse protocol while holding cells at −40 mV to bring R3 into the HCS. R2 and R3 mutations induced *I*_gp_ starting at −80 mV, highlighting a negative shift in the voltage onset of *I*_gp_ for R220G while causing a slight positive shift for both R223I and R223Q (Fig. 1C). This variable voltage-dependent onset of *I*_gp_ further indicates that R2 occupies the very constriction of the gating pore at the more negative potentials. Conversely, R3 secures the gating pore at more depolarized potentials. We also detected a difference between the R223Q and R223I mutant in the amplitude of the *I*_gp_, with R223Q exhibiting a higher *I*_gp_ beginning at membrane potentials as negative as −140 mV (Fig. 1C). This result suggests that, even at the same residue, the specific nature of the amino acid substitution can influence the amplitude of the *I*_gp_.

Since R3 mutations in Na_V_1.4 and Na_V_1.5 were previously shown to induce outward *I*_gp_ (11, 15–17), we tested if the R223I and R223Q mutations induce outward *I*_gp_ using an intracellular solution containing 140 mM KF and an extracellular solution containing 140 mM NMDG that is known not to permeate through the gating pore in Na_V_1.4 (21), Na_V_1.5 (15), or K_V_7 (26) channels. Pulses were applied from +50 mV to −100 mV in 10-mV increments for 20 ms in cells held at −40 mV. Na_V_1.2WT and all three mutants showed similar inherent nonspecific leaks between −10 and +50 mV, suggesting no outward *I*_gp_ conducted by R223I or R223Q mutants (Fig. 1D).

Collectively, these results suggest that, albeit with different amplitudes and voltage dependence, *I*_gp_ induced by R220G, R223I, and R223Q mutant channels may affect the excitability of neurons since they are induced at a voltage range overlapping with the most relevant voltage range for neuronal firing between −40 and −100 mV (33). However, the extent of neuronal excitability alteration, as well as the differential impact on distinct neuronal subtypes, may vary across these mutations.

### Effects of Ca^2+^, Mg^2+^, and K^+^ on gating pore currents

Several studies in different populations have reported that autistic children have up to 60% reduced Ca^2+^ and Mg^2+^ concentration in blood serum compared to healthy children (34–37) (for a review, see Ref. (38)). Other studies revealed a 30–40% reduction in serum Ca^2+^ and Mg^2+^ levels in patients with epileptic seizures and status epilepticus (39, 40). Moreover, higher serum Mg^2+^ concentrations were associated with a reduced risk of overall epilepsy, with genetically determined serum Ca^2+^ levels showing an inverse association with the risk of generalized epilepsy (41). Notably, antiseizure medications such as oxcarbazepine and carbamazepine are shown to reduce Ca^2+^ and Mg^2+^ concentrations, possibly contributing to their depletion in individuals with epilepsy (39, 42, 43).

Our previous study demonstrated that the amplitudes of *I*_gp_ induced by R1 and R2 mutations in domains II and IV of the Na_V_1.2 were increased by the absence of Ca^2+^ and Mg^2+^ from the extracellular solution (24). Moreover, increased Ca^2+^ and Mg^2+^ concentrations blocked *I*_gp_. Here, we found that removing both (Ca^2+^ and Mg^2+^) cations from the extracellular solution increased *I*_gp_ amplitudes by ∼6, 10, and 10 folds for the R220G, R223I, and R223Q mutants, respectively, at −200 mV (Fig. 1E). These results show that reducing external Ca^2+^ and Mg^2+^ concentrations substantially increases ion flux through mutant gating pores, primarily at negative membrane potentials, which can exacerbate epileptic and ASD symptoms in patients with low Ca^2+^ and Mg^2+^ levels. Interestingly, R223Q conducted significantly higher *I*_gp_ amplitude than R223I at negative potentials, confirming that the identity of the substituting residue influences gating-pore size. Compared to glutamine (Gln), isoleucine (Ile) is more hydrophobic and has a bulky, nonpolar, hydrocarbon side chain.

Previous studies showed that gating pores are nonselective (7, 13, 26) and thus can permeate other ions, such as K^+^ and Cs^+^ (7, 13). By using an extracellular solution containing 140 mM KCl, 1 mM CaCl_2_, 1 mM MgCl_2_, and 10 mM HEPES, and holding the voltage at −80 mV, we found that the R220G, but not R223Q or R223I mutant channels, conducted K^+^  *I*_gp_ starting at −60 mV and increased at more negative voltages (Fig. S2), similar to the Na^+^  *I*_gp_ (Fig. 1B). Given the abundance of Na^+^ in CSF, we hypothesize that the inward *I*_gp_ mediated by Na^+^ in R220G may be a key factor in disrupting the resting membrane potential and firing of action potentials. However, when the membrane potential becomes more negative than the potassium equilibrium potential (*E_K_*), the electrical force becomes strong enough to overcome the concentration gradient, pulling potassium ions into the neuronal cell.

### Voltage dependence of activation, inactivation, and recovery from inactivation of the Na_V_1.2 mutants

**Figure 2:**
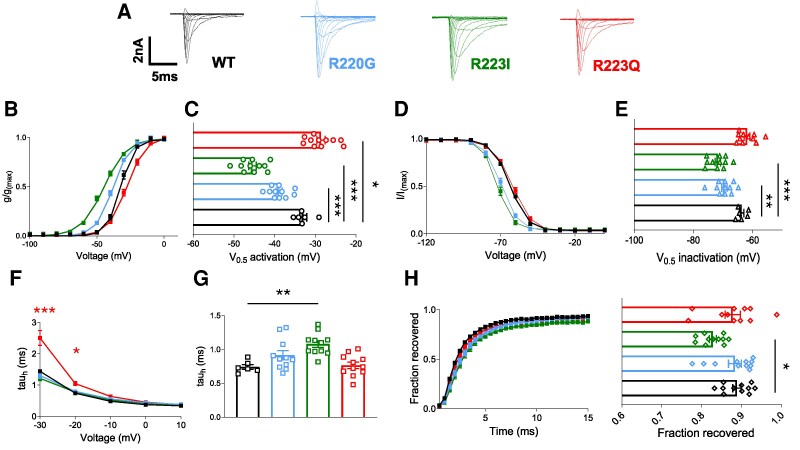
Voltage-dependent activation, inactivation and recovery from inactivation of Na_V_1.2 mutants. A) Representative families of current traces elicited by voltage steps from −100 to +40 mV from a holding potential of −120 mV induced by human Na_V_1.2WT, R220G, R223I, and R223Q. B, C) Voltage-dependent steady-state activation curves show a significant hyperpolarizing shift in Na_V_1.2(R220G) and Na_V_1.2(R223I) mutants and a depolarizing shift in Na_V_1.2(R223Q). D, E) The voltage dependence of inactivation was determined using 100-ms conditioning pulses to various potentials ranging from −120 to +70 mV in 10 mV increments. The remaining sodium current was subsequently elicited via a 20 ms long test pulse at −20 mV. Voltage-dependent steady-state inactivation curves indicate a significant hyperpolarizing shift in the inactivation of Na_V_1.2(R220G and R223I), while R223Q showed a similar inactivation pattern to Na_V_1.2WT. F) To determine the time constants of fast inactivation (*t_h_*), a second-order exponential function was fit to the time course of fast inactivation from the peak current at each pulse to 20 ms after the onset of the depolarization, yielding two time constants, with only the fast one used (*t_h_*). Mean values for *t_h_* as a function of test pulse voltage. Na_V_1.2(R223Q) exhibited increased *τ_h_* at −30 and −20 mV. G) Means of t_h_ at the voltage showing the peak central pore current (depending on the nature of mutation). Only Na_V_1.2(R223I) revealed a significantly increased *τ_h_* at the peak current. H) Recovery from fast inactivation at −120 mV. Na_V_1.2(R223I) decreases the total fraction recovered compared to Na_V_1.2WT. *n* = 6, 11, 11, and 11 for Na_V_1.2WT, R220G, R223I, and R223Q, respectively. One-way ANOVA followed by Dunnett's post hoc test for multiple comparisons was used. Error bars indicate SEM; * indicates *P* < 0.05; ** indicates *P* < 0.01; *** indicates *P* < 0.001. The extracellular patch-clamp solution contained:140 mM NaCl, 2 mM CaCl_2_, 2 mM MgCl_2_, and 10 mM HEPES.

R220G, R223Q, and R223I mutants showed different clinical phenotypes. The distinct amplitudes and voltage dependencies of *I*_gp_ may partially explain the diverse spectra of these clinical phenotypes. To get the complete picture of the biophysical properties of these mutant channels that would have additional effects on the channel function, we employed different protocols in HEK293 cells, conducting central pore current amplitudes of *<*6 nA (Fig. 2A). Both R220G and R223I showed ∼−7 and ∼−14 mV hyperpolarizing shifts in the *V*_0.5_ of the conductance/voltage (G/V) curve, highlighting a possible gain-of-function effect (Fig. 2B, C, Table S1). In contrast, R223Q exhibited a marginal depolarizing shift in activation pattern (+3 mV) (Fig. 2B, Table S1). We measured steady-state fast inactivation by applying 100-ms conditioning pulses to various potentials ranging from −120 mV to +70 mV in 10 mV increments, followed by a 20-ms long test pulse at −20 mV. R220G and R223I showed significant hyperpolarizing shifts in fast inactivation, suggesting a loss-of-function effect, whereas no change was observed for R223Q (Fig. 2D, E, Table S1).

The time constant of fast inactivation, *τ_h_*, representing the transition from the activated to the fast-inactivated state, was plotted versus voltage in Fig. 2F. All three mutants showed substantial voltage dependence of *τ_h_*, with R223Q exhibiting a significant slowing of τ_h_ at −30 and −20 mV (Fig. 2F, Table S1). Since the three mutations showed distinctive shifts in the voltage dependence of activation, we investigated *τ_h_* at a normalized test potential setting. Mean values of *τ_h_* at the voltage of the peak current amplitude of each mutant showed that only the R223I mutant channel significantly delayed the transition from the activated to the fast-inactivated state (Fig. 2G, Table S1).

Recovery from fast inactivation was evaluated by paired depolarization steps with different time intervals (Fig. 2H, Table S1). First-order exponential functions with an initial delay were well fit to the recovery time courses, indicating that only R220G significantly delayed the recovery from fast inactivation, *τ*_rec_, whereas R223I was the only mutant channel to decrease the fraction of recovered channels compared to the Na_V_1.2WT channel (Fig. 2H, Table S1).

Combined with the results from voltage-dependent *I*_gp_, these findings suggest that both R220G and R223I mutations exhibit varying degrees of a mixture of gain- and loss-of-function effects, while R223Q showed mainly loss-of-function effects. These effects will presumably alter the firing of different neuronal types, affecting brain circuits in individuals with epilepsy and ASD.

### The molecular basis of eliciting gating pore currents

**Figure 3:**
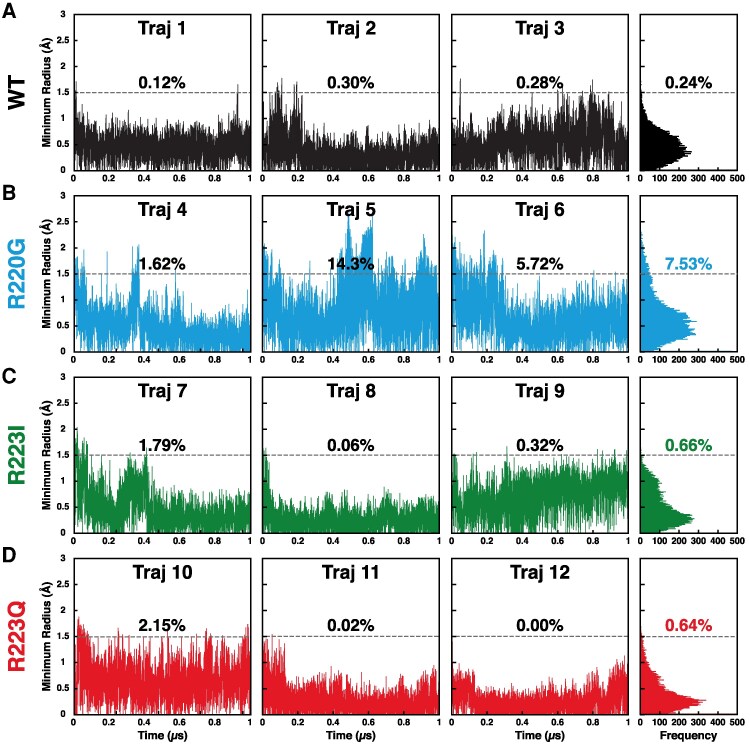
Pore opening of VSD_I_ mutants during MD simulations. Time series of the minimum radius of the aqueous pathway of gating pore through the VSD during trajectory simulations. Each panel represents the minimum pore radius over 1 μs for different systems: WT (A, black), R220G (B, blue), R223I (C, green), and R223Q (D, red). A 1.5 Å threshold (dashed line) is indicated as a potential criterion for water and ion permeation. The rightmost panels show the histogram of minimum radius of gating pore, representing collective frequency of the gating pore-opening events among three independent runs.

Functional studies of the three pathogenic mutations showed differential impacts on the DI Na_V_1.2 VSD. R220G showed a 4- to 5-fold bigger *I*_gp_ amplitude than R223Q/I at physiological conditions. It also shows a rightward shift in the voltage onset of *I*_gp_ compared to R223Q or R223I. Three questions guided our investigation: First, what factors stabilize S4 in a nonconducting (sealed) position? Second, what molecular interactions govern *I*_gp_ amplitude? Third, why does substituting the third arginine with glutamine (Q) produce a different effect than replacement with isoleucine (I)? To address these questions and thus assess the impact of gating-charge mutants on *I*_gp_ formation through VSD_I_, we performed multiple independent 1-μs MD simulations for WT and mutants (Fig. 3). All simulations were initiated from the same down-state conformation under identical conditions, including applying a −200 mV electric field to mimic the polarized membrane potential. The minimum radius of the gating pore permeation pathway within the VSD was monitored in all simulations. While WT maintains a tightly closed gating pore, specific mutations, particularly at R2, destabilize the pore and increase aqueous permeability through the gating pore.

WT simulations revealed a consistently sealed VSD_I_ structure with minimal pore-opening events. The minimum pore radius of WT (trajs 1–3) rarely exceeded the 1.5 Å threshold required for water molecule and ions passage, with open conformations occurring in only 0.23% ± 0.10% of frames across three independent replicas (Fig. 3A, black traces; Table S3). In contrast, the R220G mutation (trajs 4–6) dramatically altered this profile, inducing significant pore dilation across all replicas. Open gating pore conformations (radius >1.5 Å) were detected in 7.21% ± 6.47% of frames (∼31-fold over WT) (Fig. 3B, light blue traces; Fig. S3). This substantial pore dilation results from substituting a critical positive charge at the R2 position with a glycine, creating space for a water-accessible, leak-prone pathway.

The R223I mutation (trajs 7–9) showed a moderate pore-opening increase (0.72% ± 0.93%), though less dramatically than R220G (Fig. 3C, green traces; Fig. S3). The substitution of arginine with hydrophobic isoleucine disrupts the native electrostatic network, leading to moderate leak. The R223Q mutation (trajs 10–12) showed a similar mean opening frequency (0.72% ± 1.24%) as R223I, despite glutamine's ability to maintain hydrogen bonding (Fig. 3D, red traces; Fig. S3). These simulation results are consistent with the spectrum of *I*_gp_ magnitudes observed in electrophysiological studies of channelopathies. The computationally observed hierarchy of gating pore-opening probabilities (R220G >> R223Q ≈ R223I > WT) aligns with electrophysiological measurements of *I*_gp_.

**Figure 4:**
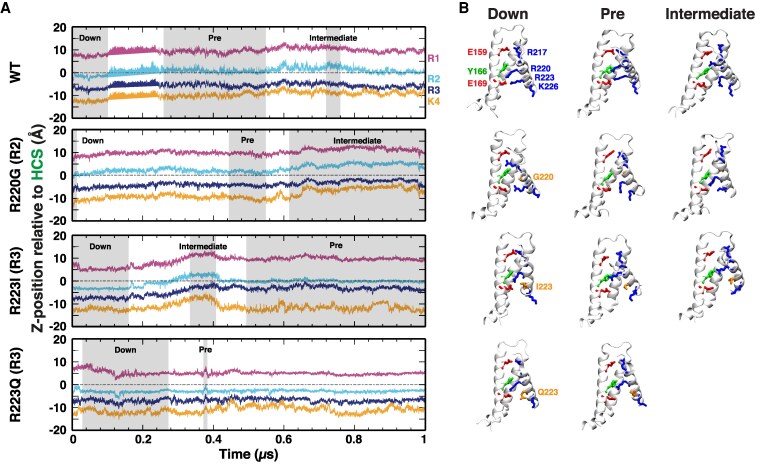
Differential dynamics of VSD_I_ gating charges across conformational states in WT and mutant Na_V_1.2 channels. A) Z-position traces of the Cα atoms of gating-charge residues relative to the HCS (Y166) over 1 μs of simulation under an applied electric field of −200 mV. Each panel corresponds to a representative trajectory for WT (Traj 2), R220G (Traj 4), R223I (Traj 7), and R223Q (Traj 10). Gray shaded regions denote time intervals assigned to each labeled conformational state (down, pre, or intermediate) based on the z-position of the gating charges relative to Y166 (see Materials and methods). B) Molecular representations of VSD_I_ in the Down, Pre, and Intermediate conformational ensembles for WT, R220G, R223I, and R223Q. VSD_I_ is shown in white cartoon representation. Gating-charge residues (R217, R220, R223, and K226) are depicted as blue licorice; the HCS residue Y166 is shown in green; acidic counter-charge residues E159 and E169 are shown in red; and mutant side chains (G220, I223, and Q223) are highlighted in orange. Conformational ensembles were computationally inferred from simulation trajectories and do not represent experimentally resolved structures.

Having established significant differences in gating pore formation, we investigated how gating-charge mutations affect VSD structural stability and facilitate gating pore formation. The structural dynamics of the VSD_I_ were analyzed by tracking the z-position of gating charges (R1/R217, R2/R220, R3/R223, and K4/K226) relative to HCS/Y166 throughout all simulations, with representative molecular views of each conformational ensemble shown alongside the corresponding z-position traces (Fig. 4). In the WT simulation, gating charges maintained relatively stable positions with only minor fluctuations around their initial coordinates, preserving the channel in the down-state structure. In this down-state ensemble, R217 (R1) and R220 (R2) are positioned above the HCS while R223 (R3) and K226 (K4) remain below, maintaining well-coordinated interactions with the acidic countercharges E159 and E169 (Fig. 4B).

In contrast, the R220G (R2) mutation resulted in a noticeable upward displacement of R2, leading to a destabilization of native electrostatic interactions of the down state and a shift toward an intermediate conformation. The R223I (R3) mutation induced an upward shift of gating charges (R1 to R3), positioning them above the down-state configuration but below the intermediate state observed in the R220G mutant—referred to hereafter as the “pre” state (Fig. 4B). This movement, coupled with R3 displacements, contributed to transient gating pore-opening events. In contrast, the R223Q (R3) mutation exhibited behavior closer to WT than that of other mutants, albeit with occasional gating pore-opening events, while R2 remained below the HCS (Fig. 4).

Our findings demonstrate that the structural and functional impacts of gating-charge mutations are highly dependent on structural context. The mutation-induced VSD conformational fluctuations strongly correlate with gating pore formation, suggesting that VSD impermeability is maintained through a network of state-dependent interactions.

### The state-dependent interaction network explains the differential mutational effects

**Figure 5:**
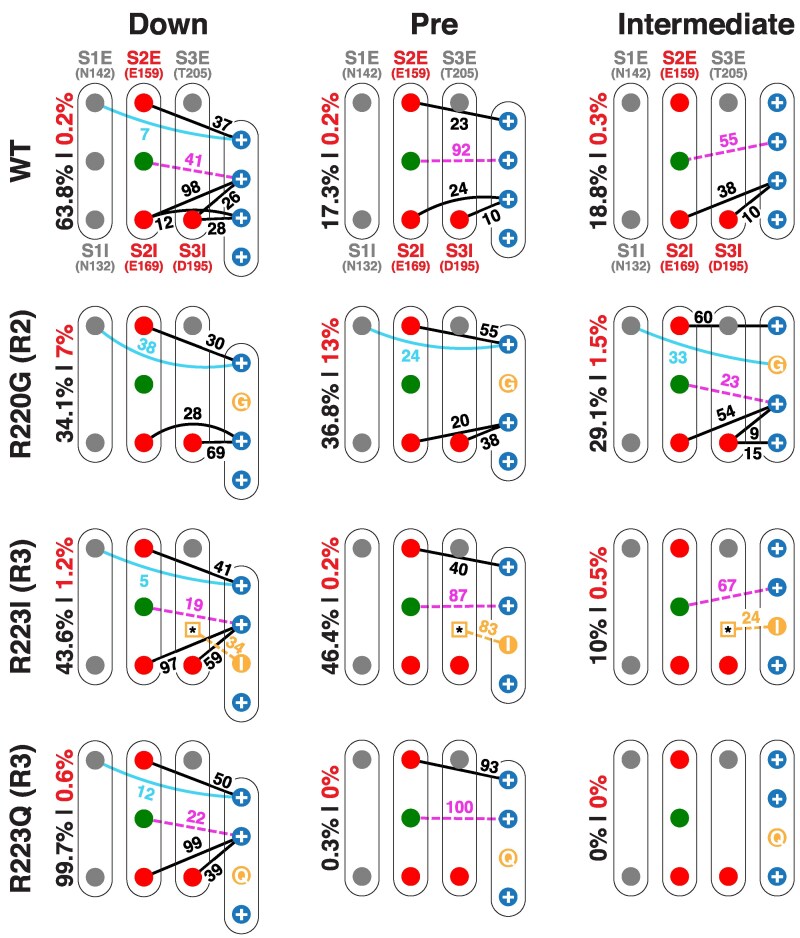
State-dependent interaction network analysis for gating charges mutants in VSD_I_. State-dependent comprehensive interaction map with occupancy of WT and mutants. This figure illustrates the key interactions observed in different structural states (down, pre, and intermediate) of the WT and mutant systems. The percentage values in black represent the proportion of time the system spends in each state during simulations, while the percentage values in red indicate the occurrence of gating pore opening within that state. Black lines: salt-bridge interactions. Light blue lines: hydrogen bond interactions. Purple dashed lines: cation–π interactions. * Orange dashed lines: collective hydrophobic interactions between I223 and residues W191, L194, and V198. Interactions <5% are not illustrated. ** State labels (down, pre, and intermediate) refer to MD-derived VSD_I_ conformational ensembles defined by the z-position of R220 relative to the HCS residue Y166 (see Materials and methods) and do not represent distinct experiment.

To further elucidate the structural basis for the differential *I*_gp_ current observed among the gating-charge mutants, we analyzed the state-dependent interaction network within VSD_I_ across different conformational states (down, pre, and intermediate) for WT and mutant channels (Fig. 5). This analysis provides insights into how key cation–π, electrostatic, and hydrophobic interactions contribute to the conformational stability of VSD_I_ and how their disruption facilitates pore opening (Figs. 4B and 5).

Cation–π interactions, particularly between gating charges and HCS, are the most critical stabilizing forces against gating pore formation. While these interactions were maintained to varying extents across all systems, R220G exhibited a complete loss of the cation–π interaction between gating charges and HCS in the down and pre state, highlighting its essential role in sealing the gating pore. This disruption resulted in high pore-opening frequencies in down and pre states (7 and 13%, respectively) (Fig. 5). In contrast, WT, R223I, and R223Q mutants retained cation–π interactions in these two states (Fig. 5), explaining their more restricted gating pore leakage compared to R220G. Interestingly, when R220G adopted the intermediate state, a compensatory cation–π interaction formed between R3 and HCS, significantly reducing leaky conformations (1.5%) (Fig. 5). This suggests that while the loss of the R2-HCS cation–π interaction increases *I*_gp_, alternative cation–π interactions in different conformations can partially limit aqueous pore formation.

Salt-bridge interactions constitute another crucial stabilizing force within VSD_I_, preventing gating pore formation by maintaining structural integrity across different conformations. The WT channel exhibits a robust network of salt bridges involving gating charges (R217, R220, and R223) and countercharge residues (E159, E169, and D195). This interaction network prevents gating pore hydration; combined with the cation–π interaction, it results in minimal occurrence of gating pore opening (0.2% opening frequency). Notably, intracellular salt-bridge interactions between gating charges and countercharges in the intracellular negative cluster (INC), E169 and D195, were well-maintained in the down state of WT, correlating with down-state occupancy of (63.8%). In contrast, the R220G mutation significantly disrupted this electrostatic network, reducing intracellular salt-bridge interactions in the down state. This destabilization weakens the electrostatic seal of the gating pore, increasing hydration and thereby increasing the pore-opening frequency.

In the R223I mutant, hydrophobic interactions involving W191, L194, and V198 are critical in maintaining VSD_I_ compaction and limiting water accessibility to the gating pore, thereby limiting water accessibility and potential ion permeation. The hydrophobic interactions between I223 and W191/L194/V198 emerged with 83% occupancy in the prestate, significantly stabilizing this structural state (46.4% of simulation time) (Fig. 5). These hydrophobic interactions reduce the electrostatic barrier and render the prestate more energetically favorable. They drove an upward displacement of the voltage sensor, as evidenced by the significant increase in prestate occupancy (46.4% compared to WT's 17.3%). Notably, these hydrophobic interactions functionally substitute for INC salt bridges, which is demonstrated by the low incidence of gating pore opening in the prestate of R223I, despite the complete absence of intracellular salt bridges.

Our state-dependent interaction network analysis uncovers a nuanced interplay of fundamental molecular interactions that govern gating pore accessibility and structural stability. To quantify the relative contribution of each interaction type, we computed both Spearman rank (*ρ*) and Pearson (*r*) correlations between interaction occupancy and the log_10_-transformed gating pore-opening probability across all simulations (Fig. S4). Cation–π interactions exhibited the strongest negative association with pore opening (*ρ* = −0.635, *r* = −0.620), indicating that increased cation–π occupancy is robustly associated with a more effectively sealed gating pore (Fig. S4A). Hydrophobic interactions also showed a moderate negative correlation (*ρ* = −0.344, *r* = −0.322), supporting their role as secondary stabilizing interactions (Fig. S4D).

In contrast, hydrogen bonds displayed a positive correlation with gating pore opening (*ρ* = + 0.550, *r* = +0.475), suggesting that increased hydrogen bonding is associated with enhanced pore hydration and likely reflects water-mediated interactions within the permeation pathway rather than structural stabilization (Fig. S4C). Salt bridges showed negligible correlation with pore opening (*ρ* = −0.042, *r* = −0.108), indicating that their contribution is minimal or highly state-dependent across the full simulation ensemble (Fig. S4B).

Collectively, these results identify cation–π and hydrophobic interactions as the primary determinants of gating pore sealing, while hydrogen bonds appear to facilitate pore hydration rather than stabilization. Salt bridges, in contrast, do not serve as reliable predictors of gating pore behavior under the conditions examined. Building upon our recent investigation of salt bridges (27), these new insights—particularly those related to cation–π and hydrophobic interactions—significantly extend our mechanistic understanding of how gating-charge mutations influence pathogenic *I*_gp_ formation and VSD structural preferences.

To evaluate the robustness of our cation–π findings to force-field parameterization, we performed an additional set of MD simulations using CHARMM36m with NBFIX corrections specifically optimized for cation–π interactions (CHARMM36m-NBF) (44). Analysis of cation–π contacts using the same geometric criteria showed that the qualitative interaction trends are unchanged; notably, cation–π interactions were more stable in CHARMM36m-NBF trajectories than in the original force field (Figs. S5 and S6). These results confirm that our conclusions regarding cation–π disruption are robust to cation–π-specific force-field corrections.S5 and

## Discussion

### Diverse clinical diagnosis of Na_V_1.2 mutants in domain I

The clinical diagnoses of the three mutations investigated in this study have previously been reported. Specifically, the R220G mutation in Na_V_1.2 was identified in a female patient with DEE (45), characterized by pharmacoresistant seizures alongside profound cognitive and neurological deficits. The onset of seizures was at 3 months and included generalized tonic–clonic seizures and infantile spasms with generalized spike and slow wave (EEG) (45). In addition to seizures, the patient suffered from global developmental delay and hyperkinetic movement disorder. For the R223I mutation, a single study reported an affected individual identified through trio-based whole-exome sequencing. This patient presented with global developmental delay, ID, and autistic behaviors as well as abnormal brain MRI findings (46). R223Q mutation in Na_V_1.2 was first reported in a Sicilian family with 17 affected individuals. Seizures were reported to begin at around 3 months in all affected infants and ceased at 12 months, consistent with the clinical diagnosis of BFNIS (47). This clinical phenotype was confirmed in another family with two individuals carrying the same mutations with focal and tonic–clonic seizures that appeared between 3 and 3.5 months, and the offset was between 12 and 14 months (48).

### Gating pore currents in Na_V_1.2 mutants in domain I

The combination of viral transduction via the BacMam system and cell cycle arrest at the G1/S boundary (30) achieved an average peak central pore current of ∼20 nA in HEK293 cells. This strong expression and current amplitudes enabled us to record *I*_gp_ in these cells. The three mutations in the Arg residues of the S4 segment of domain I of the Na_V_1.2 channel (R2: R220G, R3: R223I, and R3: R223Q) that we tested here showed distinctive *I*_gp_ amplitudes and voltage dependences. R220G exhibited the highest inward *I*_gp_ amplitude, consistent with a previous study investigating the *I*_gp_ in R2 and R3 mutations in domain I of the cardiac Na_V_1.5 channel (15). The inward *I*_gp_ induced by R220G was conducted starting from −60 mV, when cells were held at −80 mV during the acquisition protocol, compared with the more hyperpolarized onsets for the R2: R853Q mutation in domain II and R1629H mutation in domain IV, reported previously (24). The position of the substituted gating charge within the S4 segment and its local electrostatic environment likely influence voltage dependence, as each residue's contribution depends on its location within the electric field and interactions with countercharges in the surrounding S1–S3 segments (12). Differences in how deeply an S4 arginine resides within the electric field at resting versus depolarized potentials can alter the voltage at which a gating pore becomes conductive, as previously demonstrated in K_V_7.2 and K_V_7.3 channels, where R1 and R2 mutations produce *I*_gp_ with distinct voltage dependence (26). Beyond the position of the mutated residue, the unique sequence composition of each VSD domain imposes a distinct electrostatic and steric environment around S4—such that equivalent gating-charge positions experience fundamentally different counter-charge networks with S1–S3 residues (27). We previously demonstrated this principle in Na_V_1.5, where equivalent R2 mutations in domain I (R222Q) and domain II (R811Q) produced *I*_gp_ with distinct voltage dependence, confirming that domain-specific VSD context governs S4 translocation energetics, gating charges positioning relative to the HCS, and the threshold voltage at which the gating pore becomes water-accessible (27). In addition, the distinct physicochemical properties of the substituted residues may influence local packing and hydration within the VSD, potentially contributing to the observed differences in *I*_gp_ voltage dependence. Accordingly, the more positive onset observed for R220G compared with R853Q and the R1629H mutations may reflect a distinct energetic landscape for movement of this gating charge, potentially involving differences in packing of nearby residues or hydration of the gating pore pathway. Further structural interpretation, possibly informed by high-resolution models or additional mutagenesis, would help clarify the electrostatic basis of this shift.

On the other hand, the inward *I*_gp_ was reduced when cells were held at −40 mV and appeared at a more hyperpolarized voltage, suggesting that R2 occupies the HCS in the resting state of the voltage sensor at negative membrane potentials. By changing the composition of the extracellular solution, we showed that *I*_gp_ induced by the R220G mutant can be carried by both Na^+^ and K^+^, suggesting a possible role for both ions in modulating neuronal firing. We suggest that Na^+^ influx is the predominant current through such gating pores, given the high Na^+^ concentration in CSF. Still, since *I*_gp_ is increased at more negative potentials with membrane potential hyperpolarizing to near −97 mV (the K^+^ reversal potential) during the afterhyperpolarization following an action potential (49), influx of K^+^ through the gating pore can contribute significantly to the total inward *I*_gp_.

R3 mutations (R223I and R223Q) showed very small *I*_gp_ amplitudes starting at a more depolarized voltage when the cells were held at −40 mV instead of −80 mV, suggesting that R3 occupies the HCS at a more depolarized voltage. Although previous studies showed that R3 mutations in other sodium channels can induce an outward *I*_gp_ (11, 15–17), R223I and R223Q did not induce an outward *I*_gp_ in our hands. This difference likely reflects isoform-specific properties of the VSD, including variations in the HCS and the precise dynamics of S4 segment movement, which can alter the formation and conductance of gating pores across different sodium channel subtypes.

Previous studies have shown that divalent cations Ca^2+^ and Mg^2+^ diminish *I*_gp_ induced by mutations in the Arg gating charges of domains II and IV by blocking the gating pore in a dose-dependent manner (24). Here, we show that removing Ca^2+^ and Mg^2+^ from the extracellular solution caused up to a 10-fold increase in *I*_gp_ in Arg gating-charge mutations in domain I, suggesting the unique feature of divalent cations in blocking *I*_gp_ in sodium channels regardless of the tested domain. Interestingly, in the presence of Ca^2+^ or Mg^2+^, the probability of pore block by these divalent cations may be higher for R223Q than for R223I. This suggests that glutamine (Gln) may coordinate divalent ions and enhance their binding affinity, whereas isoleucine (Ile) cannot bind ions. These physicochemical properties would strongly reduce water accessibility through the gating pore, thereby limiting *I*_gp_ conductance. Notably, reductions of Ca^2+^ and Mg^2+^ concentration in serum—and potentially in CSF—have been reported in patients with epilepsy and ASD (34–37, 39, 40); however, the link between these systemic divalent reductions and increased *I*_gp_ in neurons remains to be confirmed. The increased amplitude of *I*_gp_ under low divalent conditions in heterologous cell models is consistent with the possibility that reduced Ca^2+^/Mg^2+^ levels may contribute to the pathogenic effects of these mutations in affected individuals.

### Biophysical characterization of Na_V_1.2 mutants in domain I

It has been shown that neutralizing the gating charges at positions 3 or 4 in DI in many voltage-gated ion channels results in a significant depolarizing shift in V_0.5_ of the activation curve, whereas neutralizing the gating charges at positions 1 or 2 in the same domain results in a less significant hyperpolarizing shift (15, 16, 27, 50, 51). Our results confirm these findings for two mutations: R220G (R2G) shifts the activation curve by ∼ −7 mV, while R223Q (R3Q) shifts it rightward by ∼+3 mV. The hyperpolarizing shift in the activation curve of the R220G mutation indicates that R2 occupies the HCS in the resting state, and that neutralizing or removing the positive moiety disrupts the interaction between S4 and the HCS, thereby facilitating channel opening. This explains the match between the voltage onset of the inward *I*_gp_ we measured at −60 mV and the onset of the activation sigmoidal curve at the same potential. For the R223Q mutation, one would expect the *I*_gp_ to onset at −60 to −70 mV, matching the foot of the activation curve, where the channels are closed. However, the fact that glutamine is a polar amino acid would still allow its amide side chain to interact with the electron-rich π-electrons of tyrosine's aromatic ring, thereby reducing the disruption between S4 and the HCS. This will eventually reduce the *I*_gp_ amplitude.

On the other hand, the leftward shift of the activation profile of R223I (−14 mV) indicates that isoleucine destabilized the voltage sensor at the resting state, thereby stabilizing the activated state of DI S4 (16). This means that a larger negative electric force is needed to bring the S4 segment to its resting position. This explains the higher energy barrier to eliciting *I*_gp_ in the R223I mutation compared to R223Q or R220G. Nevertheless, it is essential to note that the voltage dependence of activation in mammalian sodium channels is a complex process that involves both the movement of the VSD and its coupling to the pore domain (52). This means that the correlation between the central pore current onset and the voltage dependence of VSD movement is not directly proportional. Notably, Isoleucine will also fail to form the cation–π interactions required to prevent the voltage sensor from leaking. Still, its hydrophobic nature will reduce the likelihood of creating a leaky gating pore.

Both R220G and R223I mutations shifted the steady-state inactivation leftward significantly, while R223Q did not. At the resting membrane potential (−70 mV), this shift will place 50% of channels with either of these mutations (R220G and R223I) in the inactivated state, compared to WT, where only 20% of the channels will be inactivated. This will increase the probability of immobilizing the S4 in its inactivated state, thereby stabilizing the *I*_gp_ at hyperpolarizing potentials (11, 53). The slight difference in *I*_gp_ amplitude between R223I and R223Q does not account for the difference in their corresponding clinical phenotypes. However, the ∼17 and 10 mV differences in the *V*_0.5_ of activation and inactivation, respectively, between the two mutants partially explain the differential phenotypes.

The minimal biophysical alteration of R223Q, together with the low amplitude of *I*_gp_, may explain the less severe form of epilepsy, BFNIS. In contrast, the R223I mutation produced more pronounced shifts in activation and inactivation, suggesting a stronger perturbation of channel gating that may influence neuronal excitability differently and could be associated with the neurodevelopmental features observed in ASD. For R220G, the combination of *I*_gp_, hyperpolarizing shifts in activation and inactivation, and slower recovery from fast inactivation suggests a mixed gain-of-function/loss-of-function effect that may contribute to the severe phenotype observed in DEE. Taken together, these findings indicate that the combined impact of biophysical alterations and *I*_gp_ can skew neuronal excitability in different ways. Consistent with this interpretation, recent in vivo evidence further supports the pathogenic relevance of the combined impact of *I*_gp_ and biophysical alterations. A knock-in mouse model carrying a gating-charge mutation in Na_V_1.2(R854Q), orthologous to the human R853Q mutation at the R2 position in domain II, which has been shown to induce *I*_gp_ and a ∼50% reduction in central pore current density (24), was recently generated. This model has been subsequently characterized and displays autism-like phenotypes, including impaired social interaction, repetitive behaviors, and communication deficits (54), providing direct evidence that *I*_gp_, in combination with alterations in central pore function, can translate into complex neurobehavioral abnormalities. Consequently, different mutations at the same position of the Na_V_1.2 sodium channel may produce distinct functional effects that contribute to divergent disease phenotypes, although the precise causal mechanisms remain unresolved.

### Molecular framework of mutation-induced gating pore formation

We have previously shown that gating-charge mutations modify the structural transitions between the activated and deactivated states, eventually leaving S4 at a local energy minimum (27). Analysis of electrostatic interactions across the different states sheds light on the importance of salt bridges in facilitating a smooth transition of S4 relative to countercharge residues in S1–S3 in WT compared to mutants. Another study also showed that the hydrophobicity of some S2 residues is negatively correlated with the kinetics of the VSD movement (55). This study observed that gating-charge mutants in the VSD_I_ of Na_V_1.2 induce differential *I*_gp_ amplitudes with distinct voltage-dependence. So, we sought to analyze the molecular framework governing mutation-induced gating pore formation by investigating all plausible electrostatic (cation–π, hydrogen bonds, and salt bridges) and hydrophobic interactions. It is important to note that direct ion permeation events were not observed in the present 1-μs trajectories, likely because this timescale is insufficient to reliably sample such rare conduction events. This interpretation is supported by structural evidence from Na_V_Ab structures of gating pore mutants, in which water-filled pathways with r_min ≥ 1.5 Å were shown to enable ion permeation (13), and by our previous Na_V_1.5 simulations (27) in which concurrent gating pore opening and Na^+^ permeation were observed in 5-μs trajectories.

Analysis of the state-dependent interaction network within VSD_I_ across different conformational states for WT and mutant systems demonstrated that cation–π interactions between S4 gating charges and the hydrophobic residue in S2 are the main forces that secure VSD from leaking. For example, the R220G mutation abolishes the cation–π interaction between R2 and the HCS, highlighting its essential role in sealing the gating pore. This disruption resulted in high pore-opening frequencies in the down and pre states. When the cation–π forces get restored at the intermediate state between R3 and the HCS, the gating pore gets sealed. In contrast, cation–π interactions were maintained between R2 and HCS in the case of R223I and R223Q mutations at all investigated states (except for the intermediate state of R223Q), which explains the low probability of inducing *I*_gp_ in these two mutations compared to R220G.

Hydrophobic interactions represent the second line of defense against VSD leaking. We found that hydrophobic residues (W191, L194, V198) in S3 form hydrophobic interactions with the R223I mutation, thereby reducing the probability of creating a gating pore compared with R223Q. Interestingly, we did not observe any hydrophobic interactions in the WT VSD. This suggests that hydrophobic interactions serve as a compensatory mechanism to offset the loss of the primary sealing forces (cation–π).

The salt-bridge interactions contribution is minimal or highly state-dependent across the full simulation ensemble. It is essential to note that salt-bridge interactions act as a catalyst, facilitating the movement of S4 between the down and up states (56, 57). As a result, mutating gating charges or the S1–S3 negative charges to noncharged residues will affect the transition rates from one state to another (55). The number and complexity of salt bridges were significantly reduced in the down (leaking) state for R220G compared to WT. This situation facilitates the induction of *I*_gp_. Despite that, R223I and R223Q showed a similar reduction in salt bridges, but hydrophobic and cation–π interactions compensated for it, leading to less leakage at the down state.

We observed that, while hydrogen bonds form in the interaction network, they contribute only slightly compared to the more dominant interaction types. In conclusion, our analysis helps predict and investigate the formation of mutation-induced *I*_gp_ in voltage-gated ion channels.

## Materials and methods

### BacMam transduction and electrophysiological recording

The pEG-BacMam constructs containing the human Na_V_1.2 (NP_001035232.1) and human β1 subunit clones were used as described in Ref. (24). Single-point mutations in the Na_V_1.2 were introduced by Biomatik Corporation, Canada. All mutants were verified by sequencing the mutated cDNA. The DNA bacterial transformation and Bacmid preparation of Na_V_1.2WT and mutants (R220G, R223I, and R223Q) were conducted as previously shown (24, 30). Details regarding BacMam transduction and electrophysiological recordings are provided in the Supplementary Methods.

### Model and simulation systems building

The Cryo-EM structure of human Na_V_1.2 (PDB: 6J8E) (58) was used as the structural template for constructing the VSD_I_ simulation system. To our knowledge, 6J8E is the only available high-resolution experimental structure of the human Na_V_1.2 α-subunit, and therefore represents the sole experimental basis for atomistic simulations of this channel. A minimal construct was built retaining VSD_I_ (residues 117–250) together with the adjacent pore domain from repeat II PD_II_ (residues 880–987), to preserve the native VSD–PD interface while limiting system size. The μ-conotoxin KIIIA and auxiliary β2 subunit present in the original 6J8E structure were excluded from the simulated construct. PD_II_ was restrained throughout the simulations; because only PD_II_ was included, its polar selectivity filter residues were exposed to the lipid environment. Three selectivity filter residues in PD_II_ were mutated to alanine. Additionally, disulfide bonds were assigned based on the PDB structure (58) between residues (912–918) and (950–959). To ascertain the correct protonation states of ionizable residues, pKa calculations were conducted employing PROPKA3 (59, 60). This resulted in a model where all residues maintained their default protonation states. Subsequently, the protein's first principal axis was aligned with the z-axis using the Orientations of Proteins in Membranes database (61). The finalized systems were embedded in a lipid bilayer composed of POPC (1-palmitoyl-2-oleoyl-sn-glycero-3-phosphocholine) and POPI (1-palmitoyl-2-oleoyl-sn-glycero-3-phosphoinositol) at a 3:1 ratio. The membrane, with a surface area of 85 × 85 Å^2^, was aligned along the z-axis and generated using the CHARMM-GUI Membrane Builder (62). The system was hydrated with a 10 Å water layer on both sides of the bilayer and neutralized with 150 mM NaCl. Prior to equilibration, the system comprised approximately 53,000 atoms with dimensions of 85 × 85 × 71 Å^3^ (Table S2).

### MD simulations

Simulations described in this work were performed using NAMD software (63) (version 2.14 or 3.0) or Desmond (64) on the specialized computational platform Anton2 (65). We used CHARMM36 (66) parameters for the protein (66–68) and lipids (69), respectively, along with the TIP3P model for explicit water molecules (70) and the associated ionic parameters with NBFIX corrections (71–73). All simulations were performed under tetragonal periodic boundary conditions to the simulation box to overcome finite-size effects and mimic bulk-like properties. The simulations were performed with a time step of 2 fs. Throughout the simulations, all covalent bonds involving hydrogen atoms were constrained using the SHAKE (74) algorithm. Electrostatic and van der Waals interactions were computed at each simulation step for maximum accuracy.

Details regarding resting state (down) model generation, MD simulations, and trajectory-based structural analyses are provided in the Supplementary Methods.

## Supplementary Material

pgag230_Supplementary_Data

## Data Availability

The numerical source data underlying the graphs and charts in this manuscript have been deposited in Zenodo and are publicly available at https://doi.org/10.5281/zenodo.20819502. Due to their large file sizes, complete uncompressed molecular dynamics trajectories are available from the corresponding author upon reasonable request. The custom scripts and simulation-related code used to process the data, analyze the simulations, and generate the figures in this manuscript have been deposited in Zenodo and are publicly available at https://doi.org/10.5281/zenodo.20819502. Details of the software versions, variables, and parameters used for the simulations and analyses are provided in the Materials and methods section.
