# Supplementary material for: Investigation and computational prediction of gating pore currents in NaV1.2 mutations across clinical phenotypes
Source: PNAS Nexus. 2026 Jul 2;5(7):pgag230. doi: 10.1093/pnasnexus/pgag230 (PMC13340926; doi:10.1093/pnasnexus/pgag230)
Supplement: pgag230_Supplementary_Data [file pgag230_supplementary_data.docx]

**Investigation and Computational Prediction of Gating Pore Currents in Na_V_1.2 Mutations Across Clinical Phenotypes**

Ahmed Eltokhi^1^, Eslam Elhanafy^2^, Jing Li*^2^, and Tamer M. Gamal El-Din*^3^

^1^Department of Biomedical Sciences, School of Medicine, Mercer University, Columbus, GA 31901, USA

^2^Department of Biomolecular Sciences, School of Pharmacy, University of Mississippi, Oxford, MS 38677, USA

^3^Department of Pharmacology, University of Washington, Seattle, WA 98195, USA

These authors contributed equally: Ahmed Eltokhi & Eslam Elhanafy

*Corresponding Author: Jing Li, ^2^Department of Biomolecular Sciences, School of Pharmacy, University of Mississippi, Oxford, MS 38677, USA, jli15@olemiss.edu & Tamer M. Gamal El-Din, Department of Pharmacology, University of Washington, Seattle, WA 98195, USA, tmgamal17@gmail.com, tamer.gamaleldin@synionbio.com

Tamer M. Gamal El-Din’s current address is SYNION BIOTHERAPEUTICS Inc., Delaware

**Supplementary Material**

**Extended Methods**

**Transduction and Expression of Na_V_1.2 WT and Mutant Channels in HEK293 Cells**. Na_V_1.2 WT and mutant channels were transduced as described in^1^. Briefly, HEK293 cells were cultured in fresh DMEM medium (Corning, 10-013-CV) supplemented with 10% FBS (VWR, 89510–186) and 1% L-Glutamine:Penicillin:Streptomycin (Gemini Bio-product, 400–110). The cell cycle arrest of HEK293 cells (ATCC, CRL-1573™) was started 24 hrs before viral transduction using 2 mM thymidine (Sigma-Aldrich, T1895-25G) (in DPBS (Gibco, 14190–136)). The transduction of Na_V_1.2WT or mutant viruses together with β1 virus was done in a ratio of 5:1, with another set of experiments using small amounts of viruses maintaining the same ratio, to achieve a current amplitude of less than 6 nA for running activation, inactivation, and recovery from fast inactivation protocols. Cells were then cultured in a 37°C incubator for 48 hrs with added 2 mM thymidine. Cells were split into single cells and allowed to settle down and attach to the surface of the dish for 24 hrs before performing biophysical recording.

**Electrophysiological Recording**. Biophysical recording and data analysis were performed with the same equipment and software as described previously^2^. Recording pipettes for measuring sodium currents were pulled from borosilicate glass to achieve initial bath resistances of 1.5–3.0 MΩ. In all experiments measuring inward I_gp_, recording pipettes were filled with an intracellular solution containing: 35 mM NaCl, 105 mM CsF, 10 mM EGTA, and 10 mM HEPES. The extracellular patch-clamp solution contained: 140 mM NaCl, 1 mM CaCl_2_, 1 mM MgCl_2_, 10 mM HEPES when measuring inward I_gp_ conducted by Na^+^ mimicking the physiological conditions in the CSF **(Fig. 1B, C, Fig. S1)**. For measuring inward K^+^ I_gp_ **(Fig. S2)**, the extracellular solution contained 140 mM KCl, 1 mM CaCl_2_, 1 mM MgCl_2_, and 10 mM HEPES. To test the effect of Ca^2+^ and Mg^2+^ **(Fig. 1E)**, the extracellular solution contained: 140 mM NaCl, and 10 mM HEPES without CaCl_2_ or MgCl_2_.

We also tested if Na_V_1.2 mutant channels conduct outward I_gp_ **(Fig. 1D)** by using an intracellular solution containing 140 mM KF, 10 mM EGTA, and 10 mM HEPES, and an extracellular solution containing 140 mM NMDG-methanesulfonate, 2 mM CaCl_2_, 2 mM MgCl_2_ and 10 mM HEPES. Activation, inactivation, and recovery from fast inactivation were tested using an extracellular solution containing: 140 mM NaCl, 2 mM CaCl_2_, 2 mM MgCl_2,_ and 10 mM HEPES and an intracellular solution containing: 35 mM NaCl, 105 mM CsF, 10 mM EGTA, and 10 mM HEPES **(Fig. 2)**. All experiments were performed at room temperature. The pH of both intracellular and extracellular solutions was adjusted to 7.4 using a CsOH solution. The osmolarity of all extracellular and intracellular solutions used in this study was adjusted using sucrose to 320 mOsm and 325 mOsm, respectively.

Before recording inward I_gp_ in transduced HEK293 cells, the peak current measurement was performed in the absence of TTX by holding the cells at -120 mV for 10 min, and current-voltage (I/V) relationships were recorded in response to voltage steps (50 ms) ranging from -100 mV to +50 mV in 10 mV increments **(Fig. S1)**. Only cells with a maximal voltage error due to residual series resistance of less than 5 mV after 90% compensation were chosen for further evaluation. Tetrodotoxin (TTX) was then added to the extracellular solution with a final concentration of 1 µM. Cells were held at either -80 or -40 mV, and pulses were applied from -200 mV to -10 mV in 10-mV increments for a duration of 20 ms **(Fig. 1B, C, E)**. To differentiate between I_gp_ and inherent nonspecific leak, linear leak subtraction was performed offline, and the average nonspecific leak current estimated by linear fitting the steady-state I-V curve from -10 to -50 mV was subtracted from the total leak current.

For outward I_gp_ experiment **(Fig. 1D)**, transduced HEK293 cells were held first at -120 mV for 10 min. Cells were then held at -40 mV while pulses being applied from +50 mV to -100 mV in 10-mV increments for a duration of 20 ms. The average nonspecific leak current estimated by linear fitting the steady-state I-V curve from -60 to -100 mV was subtracted offline from the total leak current.

In cell experiments showing a peak current amplitude of less than 6 nA due to the reduced viral concentration during transduction, the activation, inactivation, and recovery from fast inactivation protocols were applied and analyzed as described in^2^.

**Resting-state (down) model preparation**. To investigate the effect of mutations on I_gp_ at resting membrane potential, we developed a resting state (down) model of Na_V_1.2 using targeted molecular dynamics (TMD)^3^. TMD simulation was guided by two collective variables to model the transition of Na_V_1.2 from its up state (6J8E)^4^ to a resting down state. For the first collective variable, we calculated the residue-based RMSD of the VSD_I_ Cα atoms between the Na_V_1.2 model and the human Na_V_1.7 structure (7XVE), which served as the template of the target model in the down state. This Na_V_1.7 reference structure contained 11 point mutations that stabilized VSD_I_ in its down conformation^5^. For the second collective variable, we specifically targeted Cα atoms of key gating charge, counter-charge, and HCS residues (E159, Y166, E169, D195, R217, R220, R223, and K226 (Na_V_1.2 numbering)) to ensure correct voltage sensor positioning. Prior to TMD, we performed structural alignment between initial structure and targeted model by superimposing immobile S1, S2, and S3 helices (residues 130-148, 156-176, and 191-208). The TMD simulation was performed for 200 ns with a force constant of 5 kJ mol⁻¹ nm⁻² throughout the simulation.

The resulting down-state structure was then equilibrated for 2 μs of unbiased MD, and the equilibrated ensemble served as the common starting point for all WT and mutant production simulations. In this way, any differences observed between WT and mutant trajectories arise solely from the mutations and their local dynamics, rather than from differences in initial conformation or from the presence of the toxin or β2 subunit in the original cryo-EM structure.

Following 5,000 steps of energy minimization, all systems underwent a three-phase simulation protocol: (i) 1 ns NPT (constant pressure and temperature) simulation with constraints on all heavy atoms; (ii) 1 ns NPT simulation with constraints on all Cα atoms; and (iii) equilibration for 98 ns in an NPT ensemble with constraints applied to the Cα atoms of HCS (166), two gating charges (R1 and R2; 217 and 220), and PD_II_ residues (880-987) to facilitate proper hydration of solvent-exposed regions within the Na_V_ VSD_I_ and pore cavity. For all MD simulations, we maintained NPT ensemble conditions using the Nosé-Hoover Langevin piston method to regulate pressure at 1 atm and a Langevin thermostat to maintain temperature at 310 K^6,7^. The piston oscillation period was set to 100 fs with a damping time scale of 50 fs. Long-range electrostatic interactions were calculated using the particle mesh Ewald (PME) algorithm^8^. For short-range non-bonded interactions, we applied a 12 Å cutoff with a smoothing function beginning at a distance of 10 Å. After completing the initial equilibration protocol, we conducted 1 μs production simulations in the NPT ensemble for each system **(Table S2)**. For MD simulations conducted using Desmond on Anton2, a Berendsen-coupling scheme was implemented to sustain a consistent pressure of 1.0 atm. The calculation of long-range electrostatic interactions was facilitated by the k-space Gaussian split Ewald method^9^. A transmembrane potential of -200 mV was applied using the constant electric-field method implemented in NAMD, in which a uniform electric field is imposed along the membrane normal (z-axis). This method assumes a spatially uniform field throughout the simulation box, which is an approximation relative to the spatially varying field in biological membranes shaped by dielectric boundaries and ionic screening. All MD trajectories were analyzed using in-house Tcl in VMD^10^ and Python scripts.

**Analysis.** The z-position distance analysis: It was used to track the movement of key gating-charge residues (R217, R220, R223, and K226). This analysis focuses on gating charge movement relative to HCS residue Y166, delineating the boundary between the extracellular and intracellular hydrated regions of the VSDs. This analysis measures the distance along the z-direction between the center of mass of the side chain of each gating-charge residue and the center of mass of HCS. To implement this analysis, we developed a custom pipeline combining Tcl scripting for trajectory processing in VMD with Python scripts for subsequent data visualization. This integrated approach allows for continuous monitoring of each gating charge's position throughout the simulation period, precisely monitoring the structural transition of each gating charge.

State classification for VSD: To analyze state‑dependent interactions **(Fig. 5)**, simulation frames were classified into three conformational ensembles (Down, Pre, Intermediate) based on the z‑position of the R2 gating charge (R220) relative to the HCS residue Y166 in S2. It is important to note that these ensembles are defined operationally from MD trajectory data and do not correspond to distinct experimentally resolved structures. State assignment was determined as follows: (i) Down state: when the center‑of‑mass z‑coordinate of R220 lies below 1 Å relative to the Y166 plane, representing a resting conformation in which R2 resides below HCS; (ii) Pre state: when R220 lies within ±1 Å of the Y166 plane; and (iii) Intermediate state: when R220 lies more than 1 Å extracellular to the Y166 plane, representing a partially activated conformation in which R2 has transited above the HCS. For each ensemble, interaction occupancies (cation–π, salt‑bridge, hydrogen bond, and hydrophobic contacts) were computed separately for WT and each mutant, allowing direct comparison of state-dependent stabilizing forces across constructs. Representative molecular views of VSD_I_ in each conformational ensemble for WT and all three mutants are provided in **Fig. 4B**

HOLE analysis: We conducted pore radius analysis using HOLE software^11^ integrated with MDAnalysis^12,13^ to characterize and visualize the gating-pore dimensions within VSDs throughout the simulation trajectories. This analysis specifically quantified the conformational changes induced by gating-charge residue movements, providing measurements of the aqueous pathway that can potentially traverse the transmembrane region of the VSD. Based on Monte Carlo simulated annealing, the algorithm identifies optimal routes for a sphere with a variable radius to pass through the channel. For each trajectory frame, only the minimum radius along the pore axis was extracted and plotted as a function of simulation time. A minimum gating-pore radius threshold of >1.5 Å was used to classify a frame as an open or leak-prone conformation. This cutoff is grounded in the molecular dimensions of water: Orttung estimated a water-molecule radius of approximately 1.4–1.5 Å from structural and refractive-index analyses of liquid water^14^, indicating that cavities exceeding this radius are readily occupied by water molecules and therefore represent potentially water-accessible pathways through the VSD. Specifically, S1-S4 protein segments from VSD_I_ (residues 131 to 232) were selected for HOLE analysis to avoid non-native hole detections. To validate the pore, we cross-checked it using VMD. Our analysis pipeline combined the HOLE radius calculations with custom Python scripts for comprehensive data visualization and interpretation.

Cation-π interaction analysis: To assess the role of cation–π interactions in VSD stability and function, we quantified these interactions throughout the simulation trajectories. This analysis was conducted using VMD in combination with the FLexible InteRaction Tool (FLIRT) script^15^ to identify cation–π interactions, followed by a Python-based post-processing step for further analysis. Cation–π interactions between S4 gating charges (R217, R220, R223, K226) and the HCS aromatic residue Y166 were evaluated by computing distances between each guanidinium nitrogen (NE, NH1, NH2) of arginine — or the amine nitrogen of lysine — and all six carbons of the Y166 ring. An interaction was counted when all six ring carbons lay within 7.0 Å of the cationic nitrogen with a maximum inter-carbon distance variation of ≤1.5 Å. This combined criterion implicitly restricts the cation to lie within a ~120° cone projecting from the face of the aromatic ring, excluding edge-on geometries, consistent with established geometric definitions^16-18^; no angular constraint on the guanidinium plane itself was applied. Interaction occupancy was calculated as the fraction of frames satisfying these criteria within each conformational state (Down, Pre, Intermediate), as defined above.

$$Cation-\pi Interaction Occupancy \left( \% \right)=\frac{\# of frames [(cation- HCS \leq7Å) \& (C dist variance \leq1.5Å) \& (selection cone \leq120^{\circ})]}{Total \# of frames}\times100$$

Molecular interactions analysis: To investigate the molecular interactions contributing to the conformational stability and gating-pore dynamics of VSD, we performed a comprehensive analysis of salt bridges, hydrogen bonds, and hydrophobic contacts across MD trajectories. All interactions were quantified using a consistent distance-based approach implemented through custom Python scripts, enabling occupancy calculations across three conformational states: down, pre-activated, and intermediate.

Salt bridges analysis allowed us to quantify the persistence of electrostatic contacts across conformational states and assess their role in stabilizing VSD. Following the methodology previously established in our work^19^, we examined dynamic electrostatic interactions between gating charges (R217, R220, R223, K226) and countercharge residues (E159, E169, D195). Pairwise distances between terminal carbon atoms of acidic and basic side chains were calculated for each trajectory. A salt bridge was defined as formed when the distance between these atoms was ≤ 5 Å.

Hydrogen bond interactions were initially screened using the HBonds Plugin (Version 1.2) in VMD to identify candidate residue pairs between gating charges and conserved polar residues (N132, N142, T205). We then applied the same distance-based Python script to quantify hydrogen bond occupancy, using a 5 Å cutoff between terminal atoms (e.g., CZ of R217 and CG of N142). This approach accounts for side-chain flexibility and captures transient polar interactions that complement electrostatic stabilization and may influence voltage-dependent conformational transitions.

To evaluate hydrophobic contributions to VSD stability, we focused on contacts between I223 and nearby hydrophobic residues W191, L194, and V198 within the S1-S3 segments. These residues were identified through structural screening as the primary hydrophobic components proximal to the HCS. Hydrophobic interactions were defined as present when any atom from these residues was within 4 Å of I223. Using the same workflow, we quantified the frequency of these contacts to assess their role in modulating gating-pore permeability and conformational rigidity.

$$Salt bridge Occupancy \left( \% \right)=\frac{\# of frames with distance \leq5Å}{Total \# of frames}\times100$$

$$Hydrogen bonds Occupancy \left( \% \right)=\frac{\# of frames with distance \leq5Å}{Total \# of frames}\times100$$

$Hydrophobic Occupancy \left( \% \right)=\frac{\# of frames with distance \leq4Å}{Total \# of frames}\times100$

**
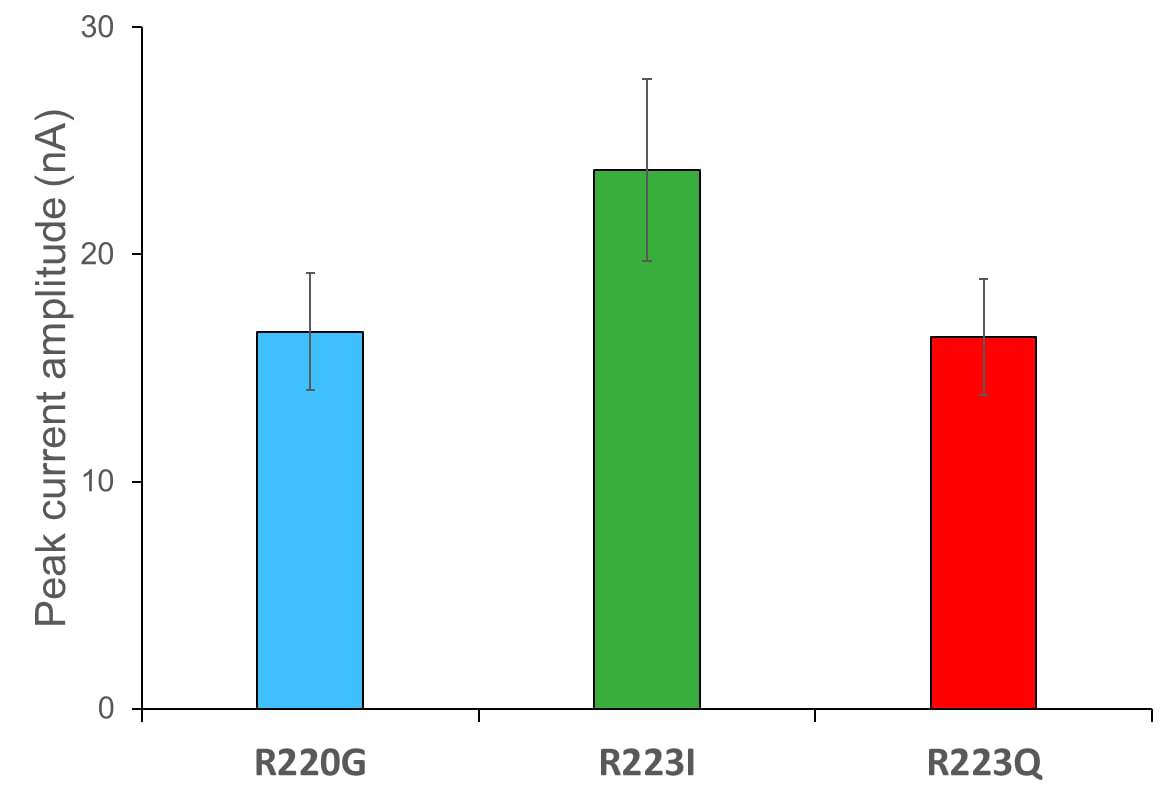
Supplementary Figures**

**Figure S1: Peak central pore current amplitudes of Na_V_1.2(R220G), Na_V_1.2(R223I) and Na_V_1.2(R223Q) mutant channels.**


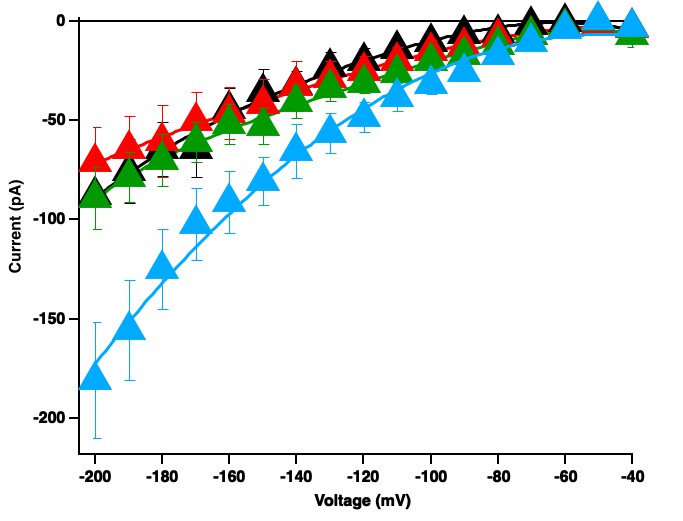
**Figure S2 Inward potassium gating pore currents (I_gp_) induced by Na_V_1.2(R220G) mutant channel.**Current–voltage (I–V) relationships of Na_V_1.2 mutants (R220G, R223I, and R223Q) and leak current of WT Na_V_1.2. Cells were held at −80 mV, and 20 ms voltage steps from −200 mV to −10 mV in 10 mV increments were applied. R220G but not R223I and R223Q induced inward I_gp_ carried by K^+^ starting at -80 mV. n= 10, 10, 9, and 8 for R220G, R223I, R223Q and WT, respectively.


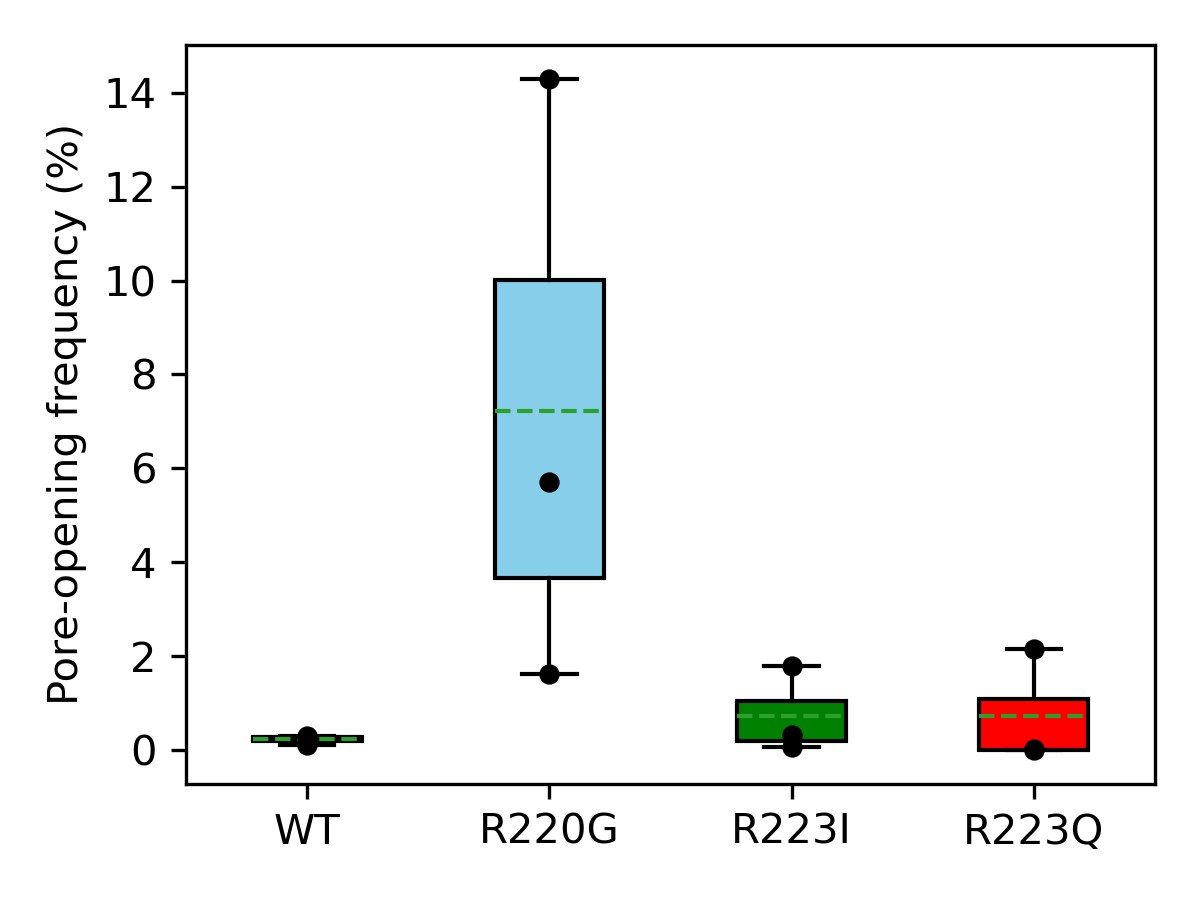


**Figure S3. Per‑trajectory pore‑opening frequencies for WT and VSD_I_ mutants.** Box plots show the distribution of pore‑opening frequencies across three independent 1‑μs trajectories for each construct (WT, R220G, R223I, R223Q). Pore‑opening frequency is defined as the percentage of frames in which the minimum gating‑pore radius exceeds 1.5 Å. Boxes indicate the interquartile range, whiskers the full range of the three replicas, and black dots represent individual trajectory values.


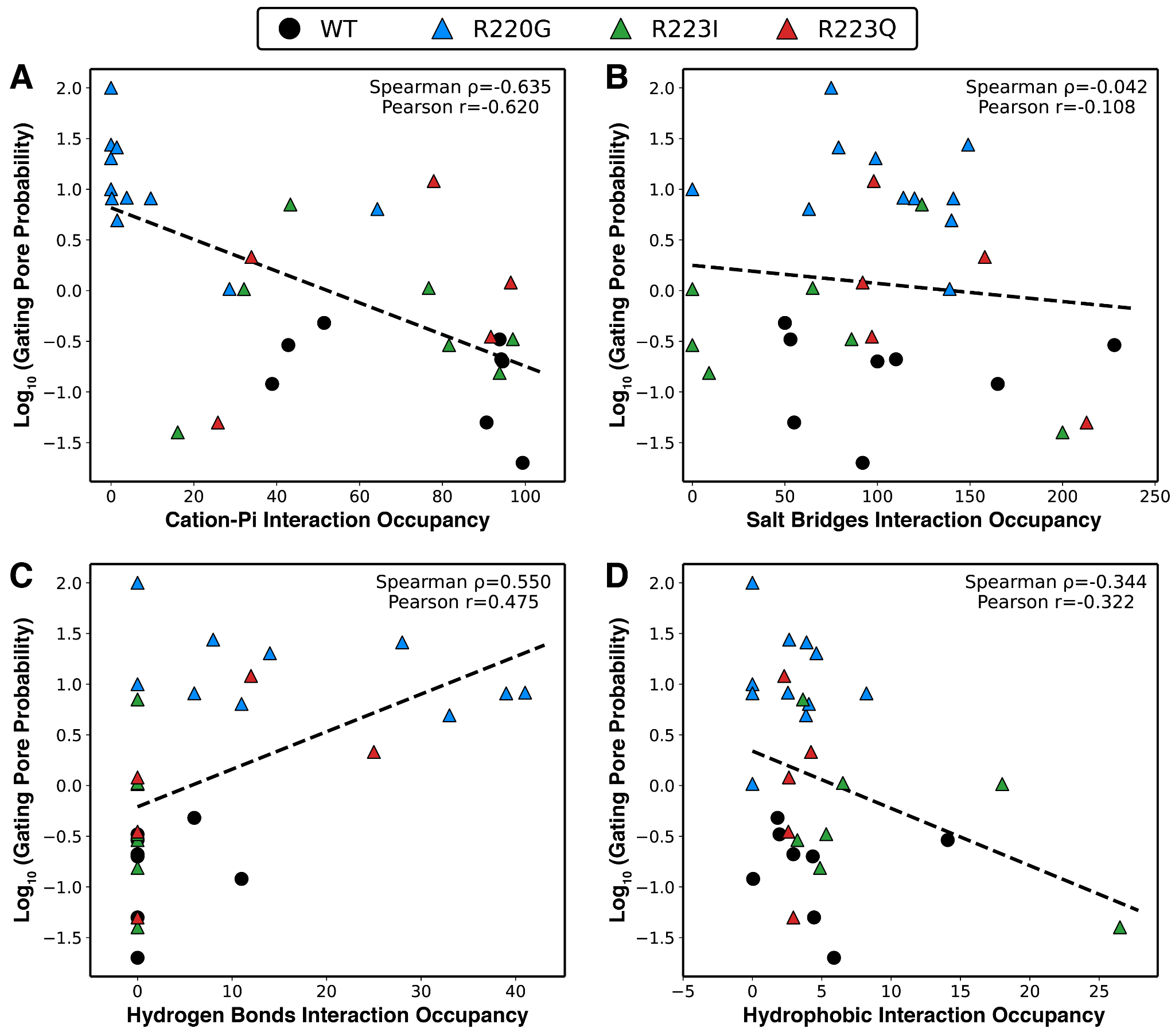


**Figure S4. Spearman and Pearson correlations between interaction occupancy and gating-pore opening probability across all simulations.** Scatter plots illustrate the relationship between interaction occupancy and the log_10_ of gating-pore opening probability for each interaction type: (A) cation–π interactions, (B) salt bridges, (C) hydrogen bonds, and (D) hydrophobic interactions. The y-axis is presented on a log_10_ scale to capture variations in gating-pore opening probability across orders of magnitude. Each point represents an individual simulation, with systems distinguished by color and marker. Dashed lines indicate linear fits. Spearman’s rank correlation coefficient (ρ) and Pearson’s correlation coefficient (r) are reported in each panel.


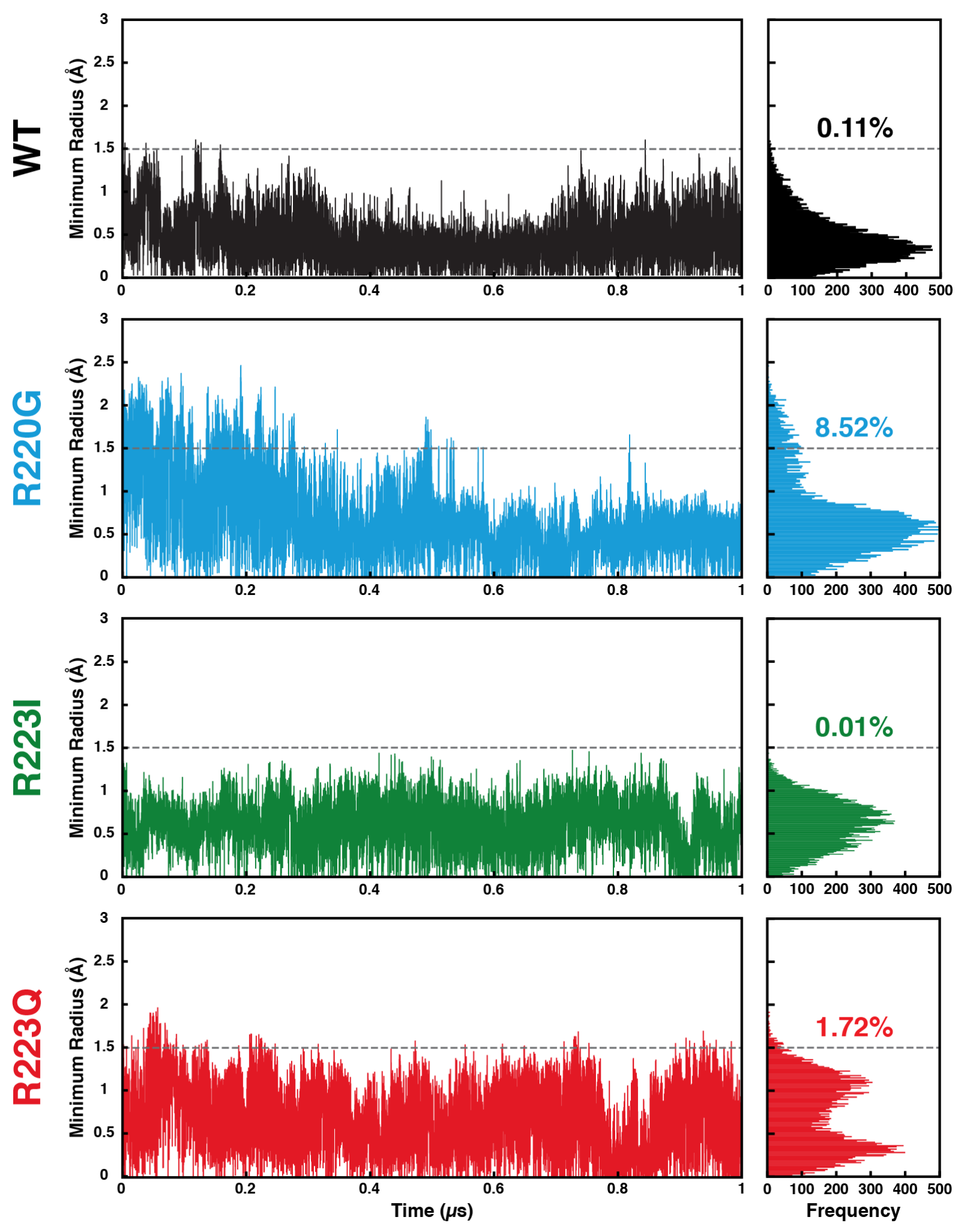


**Figure S5. Gating‑pore opening probabilities from NBFIX simulations.** Minimum pore radius as a function of time (left) and corresponding radius histograms (right) for WT, R220G, R223I, and R223Q simulated with CHARMM36m–NBFIX cation‑π corrections. The percentage in each histogram indicates the fraction of frames with minimum radius > 1.5 Å, i.e., the gating‑pore opening probability for that trajectory.


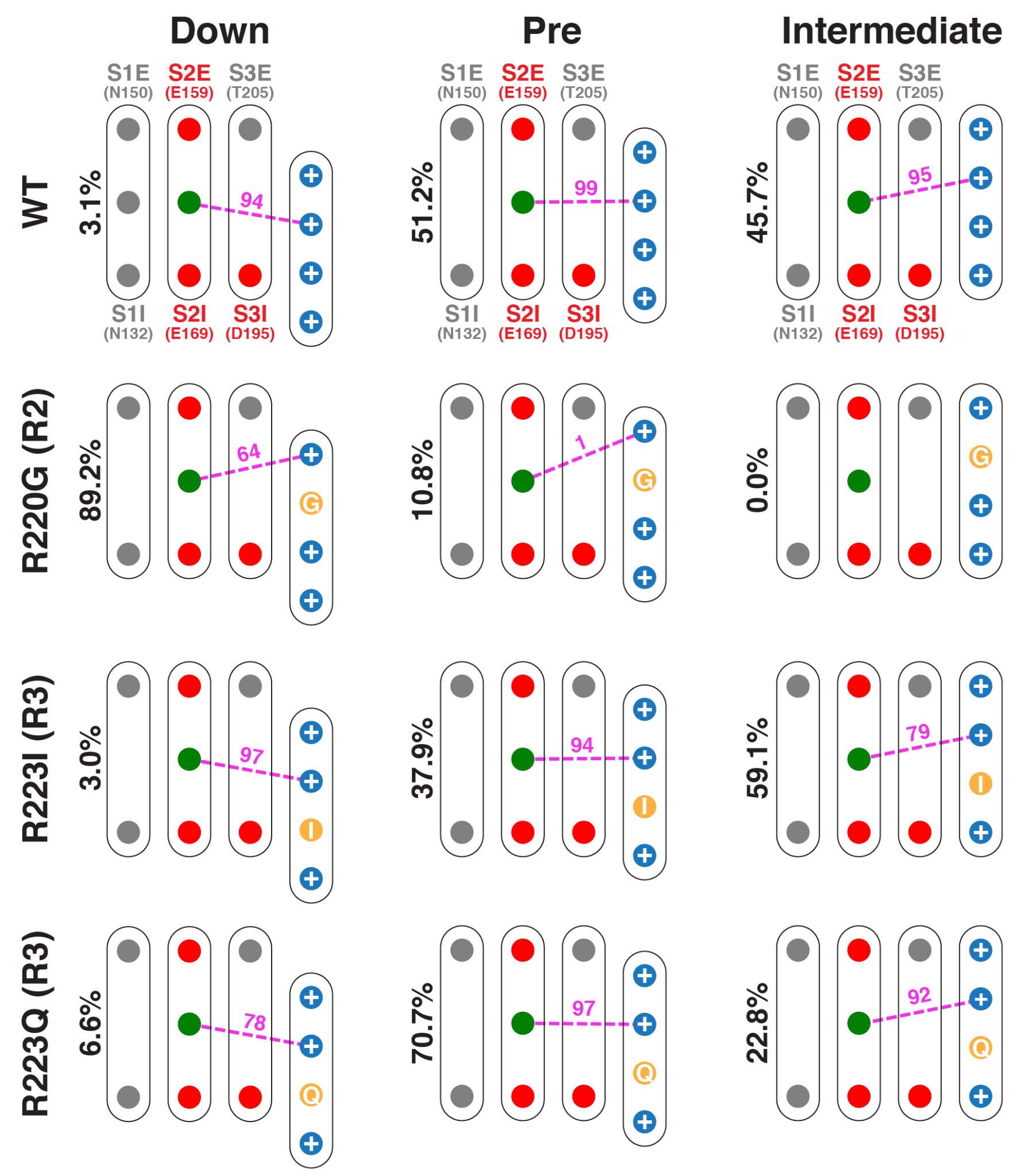


**Figure S6. State‑dependent cation‑π interaction networks with NBFIX.** Schematic interaction networks for WT, R220G, R223I, and R223Q in Down, Pre, and Intermediate VSD_I_ states obtained from CHARMM36m–NBFIX simulations. Colored nodes represent key residues (green, HCS Y166; blue, S4 gating charges; red, acidic countercharges; orange, mutant side chains). Magenta dashed lines indicate cation‑π contacts, labeled with their occupancy (% of frames) within each state.

**Table S1. Biophysical properties of Na_V_1.2WT and mutant channels in HEK293 cells.** V_0.5_ is the voltage of half-maximal activation, k_v_ is a slope factor and τ_h_ is the time constant of fast inactivation. Data are presented as means ± SEM. n = number of recorded cells. *P < 0.05, **P < 0.01, ***P < 0.001 (one-way ANOVA with Dunnett’s post hoc test or ANOVA on ranks with Dunn’s post hoc test).


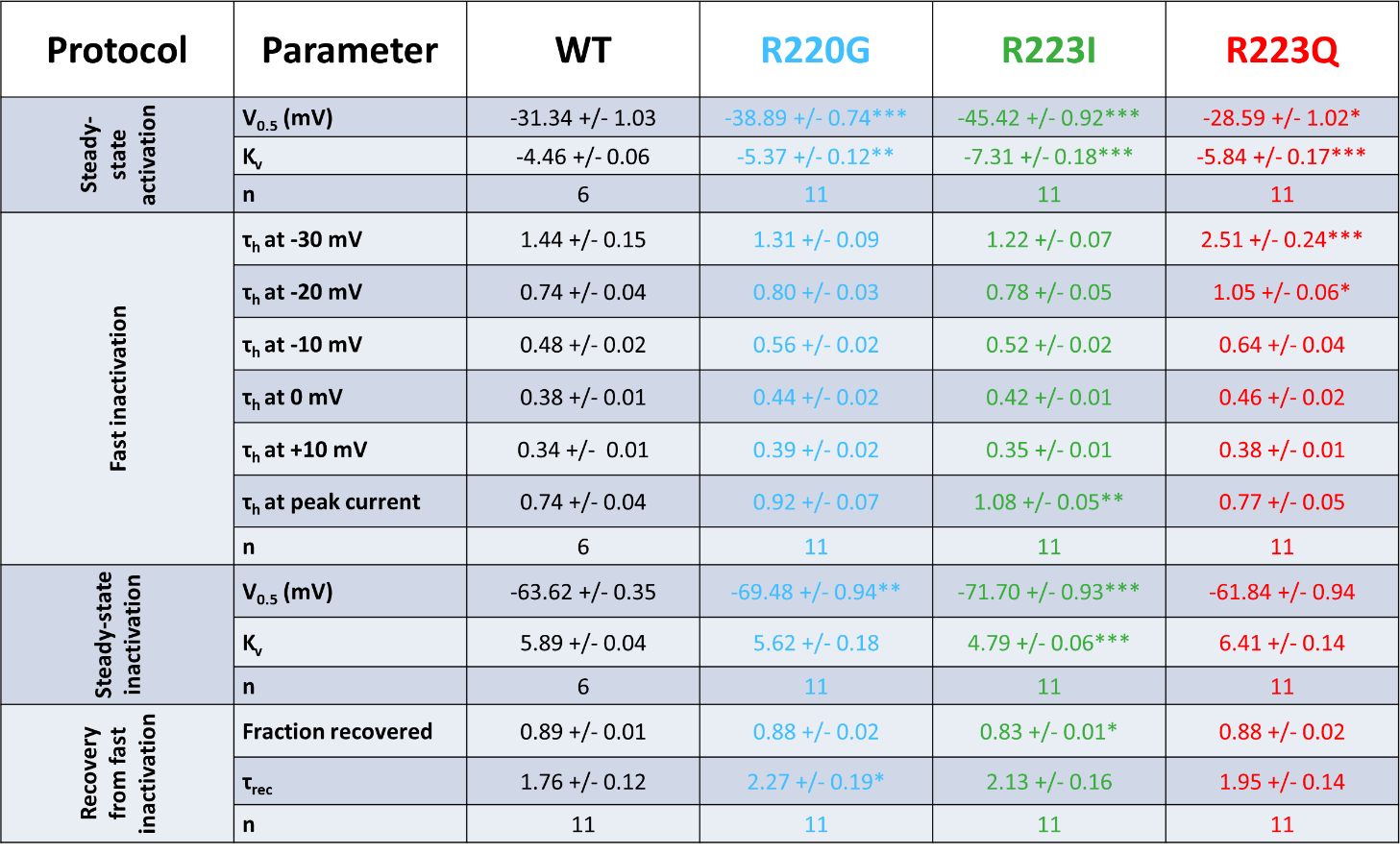


| **System** | **Composition** | **# Atoms** | **Duration (µs)** | **# of Replicas** | |
| --- | --- | --- | --- | --- | --- |
| WT | VSD_I_ (117 - 250) PD_II_ (880 - 987)  138 POPC and 46 POPI  8005 Water Molecules  NaCl [65 Na^+^ and 21 Cl^-^] | 52,821 | 1 | 3 | |
| R220G | VSD_I_ (117 - 250) PD_II_ (880 - 987)  138 POPC and 46 POPI  8004 Water Molecules  NaCl [66 Na^+^ and 21 Cl^-^] | 52,802 | 1 | 3 | |
| R223I | VSD_I_ (117 - 250) PD_II_ (880 - 987)  138 POPC and 46 POPI  8004 Water Molecules  NaCl [66 Na^+^ and 21 Cl^-^] | 52,814 | 1 | 3 |  |
| R223Q | VSD_I_ (117 - 250) PD_II_ (880 - 987)  138 POPC and 46 POPI  8005 Water Molecules  NaCl [66 Na^+^ and 21 Cl^-^] | 52,812 | 1 | 3 |  |

**Table S2: Summary of MD system composition, simulation durations, and the number of replica simulations for both WT and mutant systems.Table S3: Summary of pore-opening percentages in different trajectories for both WT and mutant systems.**

| **System** | **Traj 1** | **Traj 2** | **Traj 3** | **Mean ± SD** |
| --- | --- | --- | --- | --- |
| WT | 0.12% | 0.30% | 0.28% | 0.23% ± 0.10% |
| R220G | 1.62% | 14.30% | 5.72% | 7.21% ± 6.47% |
| R223I | 1.79% | 0.06% | 0.32% | 0.72% ± 0.93% |
| R223Q | 2.15% | 0.02% | 0.00% | 0.72% ± 1.24% |

**Supplementary references**

1 Eltokhi, A. *et al.* Pathogenic gating pore current conducted by autism-related mutations in the Na(V)1.2 brain sodium channel. *Proc Natl Acad Sci U S A* **121**, e2317769121, doi:10.1073/pnas.2317769121 (2024).

2 Eltokhi, A., Catterall, W. A. & Gamal El-Din, T. M. Cell-cycle arrest at the G1/S boundary enhances transient voltage-gated ion channel expression in human and insect cells. *Cell Rep Methods* **3**, 100559, doi:10.1016/j.crmeth.2023.100559 (2023).

3 Schlitter, J., Engels, M. & Krüger, P. Targeted molecular dynamics: a new approach for searching pathways of conformational transitions. *J Mol Graph* **12**, 84-89, doi:10.1016/0263-7855(94)80072-3 (1994).

4 Pan, X. *et al.* Molecular basis for pore blockade of human Na(+) channel Na(v)1.2 by the μ-conotoxin KIIIA. *Science* **363**, 1309-1313, doi:10.1126/science.aaw2999 (2019).

5 Huang, G. *et al.* Unwinding and spiral sliding of S4 and domain rotation of VSD during the electromechanical coupling in Na(v)1.7. *Proc Natl Acad Sci U S A* **119**, e2209164119, doi:10.1073/pnas.2209164119 (2022).

6 Feller, S. E., Zhang, Y., Pastor, R. W. & Brooks, B. R. Constant pressure molecular dynamics simulation: The Langevin piston method. *The Journal of Chemical Physics* **103**, 4613-4621, doi:10.1063/1.470648 (1995).

7 Martyna, G. J., Tobias, D. J. & Klein, M. L. Constant pressure molecular dynamics algorithms. *The Journal of Chemical Physics* **101**, 4177-4189, doi:10.1063/1.467468 (1994).

8 Darden, T., York, D. & Pedersen, L. Particle mesh Ewald: An N⋅log(N) method for Ewald sums in large systems. *The Journal of Chemical Physics* **98**, 10089-10092, doi:10.1063/1.464397 (1993).

9 Shan, Y., Klepeis, J. L., Eastwood, M. P., Dror, R. O. & Shaw, D. E. Gaussian split Ewald: A fast Ewald mesh method for molecular simulation. *The Journal of Chemical Physics* **122**, doi:10.1063/1.1839571 (2005).

10 Humphrey, W., Dalke, A. & Schulten, K. VMD: visual molecular dynamics. *J Mol Graph* **14**, 33-38, 27-38, doi:10.1016/0263-7855(96)00018-5 (1996).

11 Smart, O. S., Neduvelil, J. G., Wang, X., Wallace, B. A. & Sansom, M. S. HOLE: a program for the analysis of the pore dimensions of ion channel structural models. *J Mol Graph* **14**, 354-360, 376, doi:10.1016/s0263-7855(97)00009-x (1996).

12 Gowers, R. *et al.* *MDAnalysis: A Python Package for the Rapid Analysis of Molecular Dynamics Simulations*. (2016).

13 Michaud-Agrawal, N., Denning, E. J., Woolf, T. B. & Beckstein, O. MDAnalysis: a toolkit for the analysis of molecular dynamics simulations. *J Comput Chem* **32**, 2319-2327, doi:10.1002/jcc.21787 (2011).

14 Orttung, W. H. RADIUS AND POLARIZABILITY OF WATER FROM REFRACTIVE INDEX DATA. *The Journal of Physical Chemistry* **67**, 503-504, doi:10.1021/j100796a507 (1963).

15 Weber, D. F. InteRaction Tool (FLIRT) VMD script for summarizing interactions in MD simulations. (2019).

16 Minoux, H. & Chipot, C. Cation−π Interactions in Proteins:  Can Simple Models Provide an Accurate Description? *Journal of the American Chemical Society* **121**, 10366-10372, doi:10.1021/ja990914p (1999).

17 Petersen, F. N., Jensen, M. & Nielsen, C. H. Interfacial tryptophan residues: a role for the cation-pi effect? *Biophys J* **89**, 3985-3996, doi:10.1529/biophysj.105.061804 (2005).

18 Grauffel, C. *et al.* Cation−π Interactions As Lipid-Specific Anchors for Phosphatidylinositol-Specific Phospholipase C. *Journal of the American Chemical Society* **135**, 5740-5750, doi:10.1021/ja312656v (2013).

19 Elhanafy, E. *et al.* The differential impacts of equivalent gating-charge mutations in voltage-gated sodium channels. *J Gen Physiol* **157**, doi:10.1085/jgp.202413669 (2025).
